# Evaluation of the Effects of a Short Supplementation With Tannins on the Gut Microbiota of Healthy Subjects

**DOI:** 10.3389/fmicb.2022.848611

**Published:** 2022-04-27

**Authors:** Silvia Molino, Alberto Lerma-Aguilera, Nuria Jiménez-Hernández, José Ángel Rufián Henares, M. Pilar Francino

**Affiliations:** ^1^Departamento de Nutrición y Bromatología, Instituto de Nutrición y Tecnología de los Alimentos, Centro de Investigación Biomédica, Universidad de Granada, Granada, Spain; ^2^Area de Genòmica i Salut, Fundació per al Foment de la Investigació Sanitària i Biomèdica de la Comunitat Valenciana (FISABIO-Salut Pública), València, Spain; ^3^CIBER en Epidemiología y Salud Pública, Madrid, Spain; ^4^Instituto de Investigación Biosanitaria ibs.Granada, Granada, Spain

**Keywords:** tannins, quebracho, gall oak, gut microbiota, short-chain fatty acids

## Abstract

Western diet, high in fats and sugars and low in greens, contributes to dysbiosis of the gut microbiota, which can lead to a variety of chronic diseases related with inflammation. Supplementation with bioactive compounds can help to maintain a healthy eubiotic state. Thus, we performed a 4-weeks nutritional intervention on healthy volunteers to investigate whether a blend of natural tannin extracts could induce healthy changes in the microbial intestinal ecosystem. Changes in the composition and functionality of the microbiota could be observed from the first two weeks onward. 16S rRNA amplicon next-generation sequencing (NGS) revealed a significant increase in microbial diversity at the end of the intervention, as well as trends toward increases in the relative abundances of several beneficial taxa, such as *Ruminococcus bicirculans*, *Faecalibacterium prausnitzii*, Lachnospiraceae UCG 010, Lachnospiraceae NK4A136, *Bacteroides thetaiotaomicron* and *B. uniformis*. Remarkably, some of the identified taxa were also identified as responsible for an increase in the production of short-chain fatty acids (SCFAs), microbial metabolites that contribute to the modulation of the immune system and have various other anti-inflammatory functions in the gut. Taken together, these results suggest that the tannin supplementation could exert a prebiotic effect by selectively stimulating the growth and the activity of bacteria that are advantageous for the host.

## Introduction

In recent years, changes due to modern life behaviors have led to a progressive modification of dietary patterns (Ruel et al., 2017). In Western populations there is a tendency to consume more calories than necessary, with a huge intake of fat and a low intake of carbohydrate from cereals, vegetables and fruits. As a result, there is a low intake of natural bioactive compounds (such as polyphenols) from greens, which are of great relevance to human health (Pérez-Burillo et al., 2018; Kopp, 2019).

Moreover, an unbalanced diet together with stress and a sedentary lifestyle, characteristic of our present lifestyle, seem to have a deleterious impact on the composition and the functionality of our intestinal microbiota, which in turn has a considerable influence on our physiology (Agans et al., 2018). In particular, this situation triggers inflammatory mechanisms, including the excessive activation of innate immunity and the failure of the intestinal barrier, leading to an easy crossing of noxious microbial products and toxins (Cani et al., 2008; Martinez-Medina et al., 2014). As a consequence, microbiota dysbiosis has been causally related to the occurrence of several chronic diseases, such as metabolic syndrome or even cognitive decline, as a consequence of cross talk between the intestine and other peripheral organs (Kopp, 2019).

In view of this, attention is being focused on finding strategies to promote a healthier environment in the colonic ecosystem, in order to restore a balanced control over the immune-metabolic axis (Cires et al., 2019; Mezhibovsky et al., 2021). While pharmacological strategies have resulted mostly ineffective to this aim, several studies have reported the efficacy of polyphenol-rich diets for promoting a healthy gut environment. These bioactive compounds have a plethora of properties (e.g., antioxidant or anti-inflammatory), which help counteract chronic diseases (Molino et al., 2016; Koch, 2019). Tannins are a class of polyphenols, synthesized by plants, which have been largely described for their beneficial effects on human health, thanks to their multiple pharmacological activities (Sieniawska and Baj, 2017; Molino et al., 2019). In particular, some authors have also demonstrated, both *in vitro* and *in vivo*, the capacity of condensed and hydrolyzable tannins to interact with the gut microbiota, promoting a balance in the colonic microbial community and protecting against pathogens (Díaz Carrasco et al., 2017, 2018; Molino et al., 2021). These natural extracts can exert a prebiotic effect, through the boosting of the production of short-chain fatty acids (SCFAs), which are crucial in the maintenance of gut and immune homeostasis (Molino et al., 2018, 2021).

Short dietary interventions in healthy subjects with potentially bioactive compounds can serve as initial indicators of their capacity for modulating the gut microbiota in a positive direction (An et al., 2019; Johnstone et al., 2021). Thus, we investigated whether a supplementation of eight healthy volunteers with capsules containing a tannin blend could drive healthy modifications in the gut microbiota over a 4-week period. We employed a blend of quebracho and oak gall tannin extracts, and we investigated the effects on gut microbiota composition and the production of SCFAs, which are good indicators of the effect of a foodstuff on the intestinal microbiota.

## Materials and Methods

### Subjects and Trial Design

**FIGURE 1:**
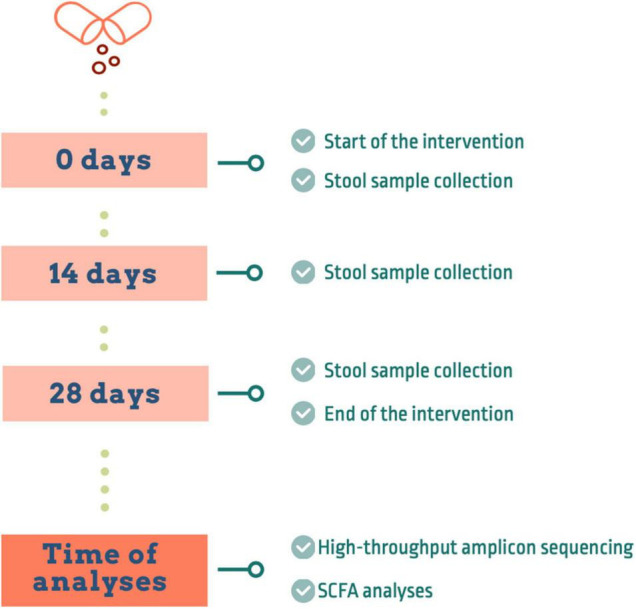
Design of the intervention study.

Eight healthy subjects were recruited at the University of Granada (Spain). Subjects were 25–45 years old and had a body mass index (BMI) within the normal range (18.5–24.99). In order to evaluate whether a short period of tannin supplementation can have a positive modulatory effect on the gut microbiota of healthy subjects, we performed a 4-weeks longitudinal nutritional intervention (Figure 1). Subjects were asked not to consume probiotics nor prebiotics during 2 weeks before the start of the trial. After that, all the participants took one capsule of tannin extract (240 mg) per day and were asked to follow an isocaloric diet. The day before starting the intervention (T0), fecal samples were taken and the sample collection was repeated after 2 weeks and at the end of the supplementation period. The trial complied with the principles of the declaration of Helsinki. The Ethics Committee of the University of Granada approved the trial protocol (1080/CEIH/2020) and informed consent was obtained from all participants.

### Dietary Supplement

Based on the results of our preliminary studies (Molino et al., 2018, 2021), two different natural tannin extracts were chosen to formulate the dietary supplement. One of the extracts is obtained from quebracho colorado (*Schinopsis lorentzii* spp.), and is characterized by the presence of condensed tannins, in particular profisetinidins (Pasch et al., 2001). The other is composed of hydrolyzable gall oak tannins, mainly represented by gallic acid and gallic acid derivatives (Radebe et al., 2013). Tannin capsules were provided by Silvateam Spa (San Michele di Mondoví, Italia), according to the formulation we suggested (two thirds quebracho extract and one third gall oak extract). The extraction methods were all food grade, characterized by a natural hot water extraction.

### DNA Extraction

Genomic DNA was extracted from bacterial suspensions obtained from stool samples, as previously described (Molino et al., 2021). Both the MagNA Pure LC JE379 platform (Roche), with DNA Isolation Kit III (Bacteria, Fungi) (REF 03264785001), and the eMAG Magnetic Extraction System (Biomerieux) (REF 418591) were used for DNA extraction, following the manufacturer’s instructions, with a previous lysis with lysozyme at a final concentration of 0.1 mg/ml. These two DNA extraction methods were previously proven to produce reliable and comparable results in our laboratory. DNA integrity was determined by agarose gel electrophoresis (0.8% w/v agarose in Tris-acetate-EDTA buffer) and DNA samples were quantified using a Qubit 3⋅0 Fluorometer (Invitrogen). All DNA samples were stored at −20°C until further processing.

### High-Throughput Amplicon Sequencing

Total DNA (12 ng) was used as template for the amplification of the V3–V4 hypervariable region of the 16S rRNA gene. PCR primers were used as described by Klindworth et al. (2013), using the forward primer (5′-TCGT CGGC AGCG TCAG ATGT GTAT AAGA GACA GCCT ACGG GNGG CWGCA-G3′) and reverse primer (5′-GTCT CGTG GGCT CGGA GATG TGTA TAAG AGAC AGGA CTAC HVGG GTAT CTAA TCC3′). For library construction, we followed the Illumina protocol for the small subunit ribosomal RNA gene (16S rRNA) Metagenomic Sequencing Library Preparation (Cod 15044223 RevA). Primers were fitted with adapter sequences added to the gene-specific sequences to make them compatible with the Illumina Nextera XT Index kit. Then, the amplicons were sequenced in an Illumina MiSeq sequencer according to the manufacturer’s instructions in a 2 × 300 cycles paired-end run (MiSeq Reagent kit v3). The data for the present study were deposited in the European Nucleotide Archive (ENA) at EMBL-EBI under accession number PRJEB46824.

### Bioinformatic Analyses

The sequence processing, assembly, Amplicon Sequence Variants (ASVs) generation and annotation were performed in the DADA2 (v1.8.0) package from R (v3.6.0) (Callahan et al., 2016). The filter and trimming parameters used were the following: maxN = 0, maxEE = c(2,5), truncQ = 0, trimLeft = c(17,21), truncLen = c(270,220) and rm.phix = TRUE. A minimum overlap of 15 nucleotides and a maximum mismatch of 1 were required for merging the forward and reverse reads. The reads were aligned using Bowtie2 (v2,3,5,1) against the human genome (GRCh38. p13) and matches were subsequently discarded (Langmead and Salzberg, 2012). The ASVs were generated by clustering sequences with 100% similarity. Taxonomic annotation was assigned by comparison to the SILVA 138 reference database using DADA2 (Quast et al., 2013). Annotation was assigned at species level for 100% similarity matches and for those matches that had a similarity of 97% or higher if there was a difference of at least 2% with the next highest match. Other sequences were annotated at the deepest possible taxonomic level.

### Short-Chain Fatty Acid Analysis

The production of acetic, propionic, and butyric acids was directly assessed in feces from the healthy donors (Delgado-Andrade et al., 2017). 100 mg of feces were weighted and resuspended in 1 ml of Milli-Q water. After centrifugation (16,000 × *g*, 2 min, 4°C), the supernatant was collected and filtered through a 0.22 μm nylon filter, and finally transferred to a vial for UPLC (Ultra High-Performance Liquid Chromatography) analysis. The samples did not require any further pre-treatment before injection. Standard solutions were quantified with concentrations ranging from 10,000 to 125 ppm.

The analysis of SCFAs was carried out on a 1290 Infinity II UHPLC (Agilent). The mobile phase was methanesulfonic acid 0.1 M pH 2.8/acetonitrile 99:1 v/v delivered at a 0.2 mL/min flow rate; the column used was an Acclaim OA C18 reverse phase (Thermo Fisher Scientific) (150 mm × 2.1 mm, 3 μm), with a total run-time of 22 min. Detection was made at 210 nm with a UV–VIS PDA detector. The results were expressed as mmol of SCFAs per Kg of feces.

### Statistical Analysis

The Shannon diversity index, Chao1 estimator and ACE were obtained with Vegan (v2.5-2) (Oksanen et al., 2012). ASVs with less than 10 counts in total were discarded. The ASV count table was normalized by total-sum scaling (TSS) in order to obtain relative taxonomic abundances. To assess the effect of tannins on the bacterial composition we analyzed the Bray-Curtis dissimilarity index between samples using Vegan (v2,5-2) and used this index for Principal Coordinate Analysis (PCoA) generated with in-house R scripts. Wilcoxon signed-rank tests with adjustment for multiple comparisons were employed to evaluate differences in richness and diversity among samples. Analysis of the composition of microbiomes (ANCOM) was used to identify differentially abundant taxa among samples and significance was determined using the Benjamini-Hochberg procedure for false discovery rate control, as described by Kaul et al. (2017). The Linear Discriminant Analysis (LDA) Effect Size (LEfSe) algorithm was applied to identify taxonomical biomarkers for the effects of tannins (Segata et al., 2011). It combines Kruskal–Wallis and pairwise Wilcoxon rank-sum tests for statistical significance assessment and feature selection. Default parameters were used for significance (*p*-value < 0.05) and linear discriminant analysis threshold (<2.0).

Correlations between gut microbiota taxonomic groups and SCFAs were performed using the network function in the mixOmics package (v6,10,9) from R, employing pair-wise similarity matrices that incorporate latent components obtained by sparse Partial Least Squares (sPLS) regression. The values in the similarity matrix can be seen as a robust approximation of the Pearson correlation (Rohart et al., 2017).

Results of SCFA production are expressed as mean values of triplicates (*n* = 3) ± standard deviation (sd). Repeated measures ANOVA with Bonferroni post-test correction was performed with the SPSS software (version 23, SPSS, Chicago, IL, United States) to determine significant differences among mean values on all the measured parameters. All the graphs were obtained using in-house R scripts.

## Results

All eight healthy volunteers completed the supplementation, taking a capsule per day over a period of 4 weeks, with a high compliance, and there were no dropouts. No adverse event was reported during the study period. To study the effect of the tannin blend on the gut microbiota, comparisons were contrasted against the baseline T0, as the study did not include any placebo supplementation.

### Effects of Tannins on Gut Microbiota Composition

**FIGURE 2:**
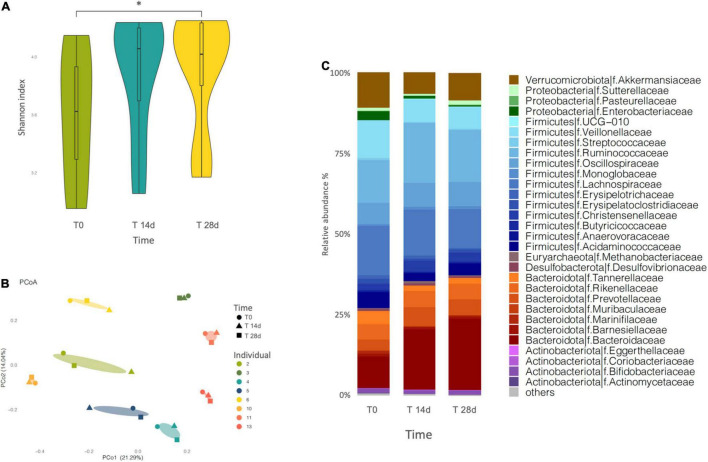
**(A)** Variation of the Shannon index of microbiota diversity throughout the intervention. Diversity increases significantly only at T28d (*p* = 0.0078, adjusted *p* = 0.016). “*” indicates the statistically significant difference. **(B)** Principal coordinate analysis (PCoA) plot of total variation based on Bray-Curtis dissimilarity of microbial genus abundance among all profiled samples. The samples cluster by individual. **(C)** Barplot of gut microbial community structure at family level, at three different times of the intervention (T0, T14d, and T28d). Relative abundances were obtained by total-sum scaling (TSS) from the genus-level abundance table.

Sequenced profiles were employed to analyze microbiome alpha diversity for each sample, based on richness estimators (Chao1 and ACE) and the Shannon diversity index (Figure 2A and Supplementary Table 1). We registered a general upward trend over the 4-week supplementation period, as estimated by all three different indexes. However, only the Shannon index showed a statistically significant difference (*p* = 0.0078, adjusted *p* = 0.016) between T0 and T28d.

We also investigated the beta diversity among the samples, calculated as Bray–Curtis dissimilarity, which describes differences in relative abundance of taxa. In the PCoA based on the Bray-Curtis dissimilarity index, PCo1 and PCo2, respectively, contributed 21,29 and 14,04% of the total variation (Figure 2B). The results did not show a separation of the samples into clusters reflecting the intervention time, rather the samples tended to group by individual. More in detail, among the different volunteers, some showed a greater difference between the samples analyzed over time (individuals 2, 4, 5, 6, and 11), while others showed almost no difference (individuals 3, 10, and 13).

At phylum level, the microbiota was dominated by Firmicutes and Bacteroidota, followed by Verrucomicrobia, Proteobacteria and Actinobacteria in all the evaluated samples. The supplementation determined a slight progressive modification in the proportions of these phyla over time, where the Firmicutes and the Proteobacteria decreased while the Bacteroidota increased, yet this trend was not significant (Figure 2C and Supplementary Table 1).

We employed ANCOM tests to determine if individual bacterial taxa changed in relative abundance during the 4-weeks intervention. We identified various genera and species that changed at a nominal level of significance, yet in no case were there statistically significant differences after adjusting for multiple comparisons (Supplementary Figures 1, 2).

**FIGURE 3:**
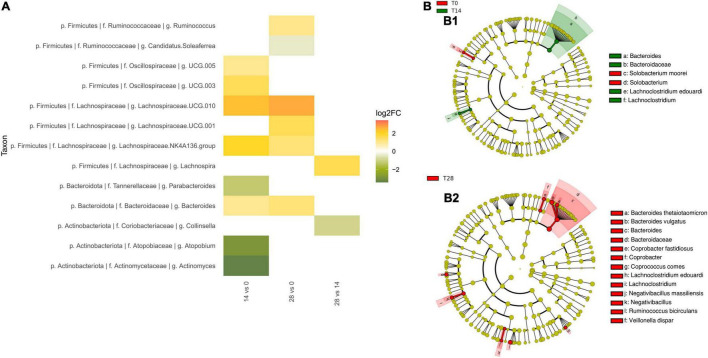
Effect of tannin supplementation on fecal microbiota. **(A)** Fold-changes in the relative abundance at genus level. All changes significant (unadjusted *p* < 0.05) at nominal level by ANCOM tests between T0 vs. T14d, T0 vs. T28d, and T14d vs. T28d are shown. **(B)** Taxonomic biomarkers characterizing the differences between **(B1)** T0 and T14d, and **(B2)** T0 and T28d according to LEfSe and represented with a cladogram visualization (*p* < 0.05, logarithmic LDA score ≥2).

Within the Firmicutes, two genus-level groups of the Lachnospiraceae family showed a nominal increase in abundance in comparisons of T0 to both time points T14d and T28d: Lachnospiraceae UCG 010 (T14d unadjusted *p* = 0.043, T28d unadjusted *p* = 0.045) and Lachnospiraceae NK4A136 group (T14d unadjusted *p* = 0.044, T28d unadjusted *p* = 0.032) (Figure 3A). Since there was no change between times T14d and T28d, the trends toward increase in these genus-level groups occurred during the first 14 days. Other taxa, such as Lachnospiraceae UCG 001, as well as the genus *Ruminococcus*, the species *R. bicirculans* and one of the ASVs of *Faecalibacterium prausnitzii*, showed trends toward increased abundance only by the end of the intervention (T28d unadjusted *p*-values *p* = 0.041, *p* = 0.027, *p* = 0.005 and *p* = 0.026, respectively). Oscillospiraceae UCG 005 and UCG 003 tended to increase slightly at T14d (unadjusted *p* = 0.033, unadjusted *p* = 0.038, respectively), but these effects were no longer present by T28d, suggesting a transitory modification in reaction to the initial exposure to the tannins.

Within the Bacteroidota, the family Bacteroidaceae (T14d unadjusted *p* = 0.041, T28d unadjusted *p* = 0.023) and the genus *Bacteroides* (T14d unadjusted *p* = 0.034, T28d unadjusted *p* = 0.013) also tended to have increased already by T14d (Figure 3A). In the case of *B. thetaiotaomicron*, the abundance of this species was highest at T28d for all individuals in the study except for one outlier (individual 3) (T28d unadjusted *p* = 0.068 with all individuals, T28d unadjusted *p* = 0.011 without outlier).

Within the Actinobacteria, the Actinomycetaceae showed a tendency to decrease in comparisons of T0 to both T14d and T28d (unadjusted *p* = 0.02 and unadjusted *p* = 0.048, respectively). Other taxa showed a trend toward decreasing only in the T0–T14d comparison, but not when T0 was compared to T28d. This was the case for *Actinomyces* (T14d unadjusted *p* = 0.034, T 28d unadjusted *p* = 0.058) and *Atopobium* (T14d unadjusted *p* = 0.025, T28d unadjusted *p* = 0.072), as well as for the family Atopobiaceae (T14d unadjusted *p* = 0.02, T28d unadjusted *p* = 0.068).

To further characterize the shifts of microbiota composition, we also conducted LEfSe analyses to identify taxa that exhibit significant differential abundance (*p* < 0.05, logarithmic LDA score ≥2) (Figures 3B1,B2). These analyses further supported the increase of *Bacteroides* and of Lachnospiraceae family members in comparisons of T0 to both T14d and T28d, as well as the increase of *R. bicirculans* by T28d. In particular, LEfSe detected the increase of the Lachnospiraceae species *Lachnoclostridium edouardi*. In addition, the comparison between T0 and T14d detected that *Solobacterium moorei* was overrepresented in the T0 group.

### Tannins Changed the Colonic Short-Chain Fatty Acid Profile

**FIGURE 4:**
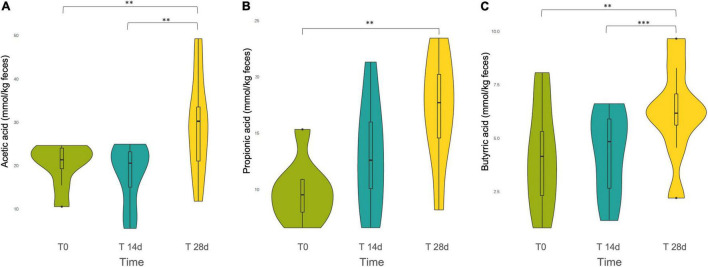
Violin plot of the short chain fatty acids (SCFAs) **(A)** Acetic acid, **(B)** Propionic acid, and **(C)** Butyric acid released during the tannin intervention, at different time points: T0, T14d, and T28d. ** and *** indicates statistically significant differences by ANOVA and Bonferroni *post-hoc* test: ^**^p < 0.01, ^***^p < 0.001.

The three main SCFAs produced in the human intestine by gut microbial fermentation, i.e., acetic acid, propionic acid and butyric acid, were quantified. According to the repeated measures ANOVA, all three SCFAs showed an increasing trend over the intervention period, but the original relative proportions among the different SCFAs present in the basal level T0 were maintained. In all cases, we found that SCFAs were released in the following order of descending abundance: acetate > propionate > butyrate (Figure 4).

Specifically, the Bonferroni *post-hoc* test evidenced that in the first two weeks the increment in the production of the different SCFAs was not statistically significant. However, the comparisons between T0 and T28d showed a statistically significant increase for acetate, propionate and butyrate (*p* = 0.001, *p* = 0.001, *p* = 0.002, respectively). Acetate and butyrate presented also a statistically significant difference between T14d and T28d (*p* = 0.001, *p* < 0.001, respectively).

### Correlations Between Short-Chain Fatty Acid Production and Bacterial Abundance

**FIGURE 5:**
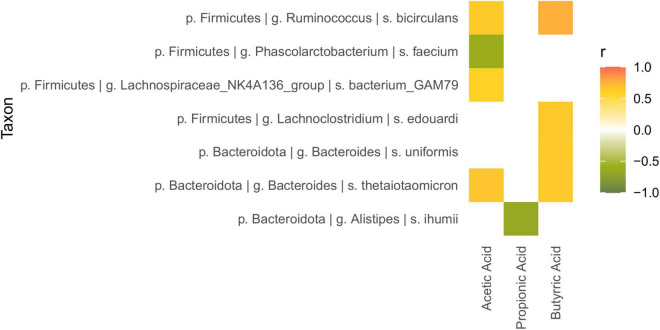
Heatmap of the correlation between microbial relative abundance and SCFA production, calculated with the sPLS-based approach implemented in the mixOmics network function (correlation coefficient >0.6).

The mixOmics network function for (sPLS) regression was used to explore the potential correlation between the gut microbiota shifts and SCFA production (Figure 5). *Alistipes ihumii* and *Phascolarctobacterium faecium* were detected to negatively correlate with propionic acid and acetic acid, respectively (correlation coefficients of −0.666 and −0.614). Lachnospiraceae bacterium GAM79, belonging to the Lachnospiraceae NK4A136 group, positively correlated with acetic acid (0.600), while *L. edouardi* and *B. uniformis* positively correlated (0.648 and 0.653, respectively) with butyric acid. Finally, the increments of *R. bicirculans* and *B. thetaiotaomicron* were associated to an augmented release of both acetate (0.6483 and 0.683, respectively) and butyrate (0.765 and 0.651, respectively).

## Discussion

Overall, the effects of the intervention on gut microbiota composition were moderate, since only the Shannon diversity index was found to increase significantly by T28d. However, it is important to consider that all participants in the intervention were healthy and lean at T0, so that their initial microbiota was likely configured in a eubiotic state. Therefore, a dietary supplementation would not be expected to produce large changes, only minor improvements. Furthermore, it must be taken into account that the supplementation lasted for a relatively short period of time, further limiting the possibility of large changes. Indeed, although some 4-week interventions with potentially bioactive compounds have resulted in substantial gut microbiota changes in healthy people, most produce positive but moderate modifications (Tzounis et al., 2011; An et al., 2019; Johnstone et al., 2021). Nevertheless, such results in short interventions with healthy subjects represent a good initial indication of the capacity of bioactive compounds for gut microbiota modulation. Similarly, in our case, the 4-week intervention was sufficient to enable us to detect some tendencies toward increases or decreases of certain taxa with tannin supplementation, mostly starting already in the first two weeks, and continuing to a lesser extent until the end of the intervention. Furthermore, we also identified a significant increase of acetate, propionate and butyrate production due to the tannin supplementation. This confirms our previous results *in vitro*, showing that both hydrolyzable and condensed tannins stimulate the production of these microbial metabolites by promoting the growth of beneficial bacteria and their metabolic functions (Molino et al., 2021). The search for evidence of SCFA production is of great relevance because these compounds exert several health-promoting functions, such as contributing to modulate cellular metabolism and immune responses (Koh et al., 2016). Remarkably, SCFA production correlated with some of the taxa that increased nominally after tannin supplementation, suggesting which taxa are actually induced by tannins *in vivo* to increase the production of these molecules.

Despite the lack of significance after correction for multiple comparisons in ANCOM analyses, we detected some trends of change in the composition of the microbiota, as stated above. Among the Firmicutes, we detected that the tannin supplementation promoted trends toward the increase of several genera belonging to the families Lachnospiraceae, Ruminococcaceae and Oscillospiraceae. All these families belong to the order Clostridiales, and include numerous bacteria involved in the regulation of the immune system and of numerous physiological metabolic functions (Raimondi et al., 2021).

The capacity of tannins for increasing the amount of Ruminococcaceae has been previously described by several authors (Díaz Carrasco et al., 2018; Molino et al., 2021). Notably, Díaz Carrasco et al. (2018) identified an increment of the genus *Faecalibacterium*, through a 30-day tannin supplementation in broilers. *Faecalibacterium* is recognized as a major source of butyrate (Louis and Flint, 2017). Analogously, after the 4-week intervention, we also detect a trend toward higher abundance of one ASV of *Faecalibacterium prausnitzii*. More in general, many taxa of the Ruminococcaceae are known for their ability to release SCFAs (Lopetuso et al., 2013; Delgado-Andrade et al., 2017; Louis and Flint, 2017; Maya-Lucas et al., 2019), including *R. bicirculans*, which we detected to increase nominally due to tannin supplementation (Wegmann et al., 2014). Moreover, the relative abundance of *R. bicirculans* correlated with the increase of acetate and butyrate. Although this species has been described as an acetic acid producer (Wegmann et al., 2014), so far there is no evidence that it produces butyrate. Nevertheless, the production of butyric acid is likely to proceed mainly from acetate in the human gut, through the CoA-transferase route. In this process butyrate is formed by transfer of the CoA-moiety from acetic acid thanks to butyryl-CoA: acetate CoA-transferase (Louis and Flint, 2009).

The Lachnospiraceae are also known for including many species able to release SCFAs (Lopetuso et al., 2013; Delgado-Andrade et al., 2017; Louis and Flint, 2017; Maya-Lucas et al., 2019), and have been previously shown to increase through the action of tannins (Díaz Carrasco et al., 2018; Molino et al., 2021). Although many members of the Lachnospiraceae have often been shown to have positive associations with health, their overall role in the gut ecosystem is controversial, since some studies have detected increases of some members of the family, including *Blautia*, *Coprococcus*, *Dorea*, and *Roseburia*, in various intra- and extra-intestinal diseases (Vacca et al., 2020). Some of the Lachnospiraceae that show a nominal increase in abundance with tannins at both T14d and T28d are known SCFA producers, such as *L. edouardi* (Adamberg et al., 2018) and the Lachnospiraceae NK4A136 group (Ma et al., 2020; Xia et al., 2021). The Lachnospiraceae NK4A136 group was also detected to increase following polyphenol treatment in ethanol-treated mice, alleviating gut-derived LPS-mediated inflammation (Xia et al., 2021). Similarly, butyrate production by the Lachnospiraceae NK4A136 group was related to a reduction of intestinal inflammatory responses and an improvement of intestinal permeability in diet-induced obese mice (Ma et al., 2020). In addition, we found that the Lachnospiraceae bacterium GAM79, one of the members of the Lachnospiraceae NK4A136 group, positively correlated with the release of acetic acid, although the Lachnospiraceae NK4A136 group has previously been correlated only with the production of butyric acid (Ma et al., 2020; Xia et al., 2021).

The Oscillospiraceae, the third Clostridiales family for which we detected trends toward the increase of several genera with tannin supplementation, have also been identified as markers of a healthy gut, since they are protein degraders, and they may take part in mucin breakdown. This process is the result of cooperation and cross-feeding among several species, which exhibit diverse metabolic capabilities (Raimondi et al., 2021). Moreover, a positive association has been reported between the Oscillospiraceae family and the amelioration of stool consistency, as well as a negative correlation with extraintestinal pain severity in irritable bowel syndrome patients (Hollister et al., 2020).

Regarding the role of Bacteroidota, it is still not clear whether an increase of the relative abundance of this phylum is associated to Western diet and obesity or to a lean phenotype and weight loss (Fabersani et al., 2021). Nevertheless, this uncertainty is likely due to the oversimplification of analyzing phylum level changes (De Filippis et al., 2016). In our study, *Bacteroides* spp., and *B. thetaiotaomicron* in particular, tended to increase after the tannin supplementation. These bacteria have been described to metabolize a large variety of oligo- and polysaccharides from plants and to produce SCFAs, inducing satiety and regulating glucose metabolism. Moreover, *B. thetaiotaomicron* has also been shown to exhibit immunomodulatory properties, attenuating intestinal inflammation and reinforcing the intestinal barrier (Béchon et al., 2020). In fact, gut inflammation has been correlated with a reduction in the abundance of *B. thetaiotaomicron* and other *Bacteroides* species (Béchon et al., 2020), reinforcing the importance of a well-balanced gut microbiota composition for human health. In our analyses, *B. thetaiotaomicron*, like *R. bicirculans*, correlated with increases of acetate and butyrate, although it has so far only been described as an acetic acid producer (Porter et al., 2018). As mentioned above, this relationship could be explained by the fact that some gut bacteria can produce butyrate from acetate through the action of butyryl-CoA: acetate CoA-transferase (Louis and Flint, 2009). Recently, *B. thetaiotaomicron* has also been investigated for its interaction in co-culture with *Phascolarctobacterium faecium* (Ikeyama et al., 2020). Even though *P. faecium* abundantly colonizes the human gut, its functional role is still to be determined. This taxon is unlikely to use carbohydrates for its development and rather uses succinate as a substrate, which is abundantly produced by *B. thetaiotaomicron*. Succinate is a known intermediate of SCFA production in the intestine and the utilization of succinate by *P. faecium* in co-culture with *B. thetaiotaomicron* resulted in the production of propionate. However, in our analyses *P. faecium* rather correlated negatively with acetate. In the case of *B. uniformis*, we detected a positive correlation only with butyrate, which has also been recently demonstrated by Fabersani et al. (2021). Moreover, these authors proposed a direct effect on the increase of TRL5 expression. The activation of TLR5 contributed to curbing the spread of the inflammatory cascade from the intestine toward peripheral tissues in obese mice (Fabersani et al., 2021).

Apart from trends toward promoting beneficial bacteria, tannin supplementation also led to a reduction in potentially pathogenic taxa belonging to the phylum Actinobacteria. *Atopobium parvulum* has been identified as a marker and possible therapeutic target in pediatric inflammatory bowel disease patients, since it is also responsible of inducing inflammation (Muehlbauer et al., 2013). Similarly to *Atopobium, Actinomyces* is part of the resident gut microbiota. However, its increment has been associated with obesity in adolescents and with colorectal cancer (Peters et al., 2016; Chierico et al., 2018). In addition, the Firmicutes species *Solobacterium moorei*, an H_2_S producer that has been associated with halitosis (Haraszthy et al., 2008), also decreased with the intervention. Finally, a suppression of the Bacteroidota species *Alistipes ihumii* by tannin supplementation was inversely correlated with the release of propionic acid. *Alistipes* is a recently described genus and its species have been mostly isolated from patients suffering from some intestinal-related pathologies. It is not yet clear whether this taxon has a leading role rather than just a bystander or co-inducer role in the observed clinical phenotypes. What is quite certain is that *Alistipes* abundance is closely related to gut dysbiosis (Parker et al., 2020).

In summary, consistent with our hypothesis, the 4-week intervention on healthy subjects suggests that tannin supplementation could induce beneficial shifts in the gut microbiota. In a short period of 28 days, we did not expect very large changes in the microbiota, as the supplemented subjects were healthy and lean. Our goal was to evaluate through a short supplementation whether tannins have the capacity to influence the environment in the colonic ecosystem in a beneficial direction, conducive to limiting the collateral damage from the Western diet, such as the onset of low-grade chronic inflammation. Our results indicate that tannins are indeed capable of producing beneficial effects by increasing microbiota diversity and SCFA production, although longer intervention times may be needed to detect significant changes in the abundances of particular bacteria. Nevertheless, the tannin mixture did produce a trend toward increasing the growth of beneficial bacteria, which can attenuate the adverse impact of inflammation through immune regulatory effects. The most interesting result is that some of the taxa, such as *R. bicirculans*, *B. thetaiotaomicron*, and *B. uniformis*, that characterized the shift to a healthier status were positively correlated with increased production of SCFAs, which are important in maintaining the wellbeing of the host. Moreover, we found that the tannin blend produced a trend toward reducing potential pathogenic taxa, correlated with IBD or obesity and colorectal cancer. The present study may have the limitations of a short intervention time and the lack of a control group, but it provides important preliminary insights for future studies. Indeed, these data will contribute to form the basis for planning a broader 3-month-long intervention, which will be conducted within the framework of the European Commission research project Stance4Health.

## Data Availability Statement

The datasets presented in this study can be found at the following link: https://www.ebi.ac.uk/ena/browser/view/PRJEB46824.

## Ethics Statement

The studies involving human participants were reviewed and approved by the Ethics Committee of the University of Granada (trial protocol 1080/CEIH/2020). The patients/participants provided their written informed consent to participate in this study.

## Author Contributions

SM, JR, and MPF designed the research. SM and AL-A conducted the experiments and analyzed data and performed statistical analyses. AL-A conducted the bioinformatic analyses. SM and MPF wrote the manuscript. AL-A and JR provided significant advice and critically edited the manuscript. JR obtained funding and coordinated the Stance4Health project. All authors contributed to the article and approved the submitted version.

## Conflict of Interest

The authors declare that the research was conducted in the absence of any commercial or financial relationships that could be construed as a potential conflict of interest.

## Publisher’s Note

All claims expressed in this article are solely those of the authors and do not necessarily represent those of their affiliated organizations, or those of the publisher, the editors and the reviewers. Any product that may be evaluated in this article, or claim that may be made by its manufacturer, is not guaranteed or endorsed by the publisher.
