# Supplementary material for: Evaluation of the Effects of a Short Supplementation With Tannins on the Gut Microbiota of Healthy Subjects
Source: Front Microbiol. 2022 Apr 27;13:848611. doi: 10.3389/fmicb.2022.848611 (PMC9093706; doi:10.3389/fmicb.2022.848611)
Supplement: Supplementary file 1 [file Data_Sheet_1.PDF]

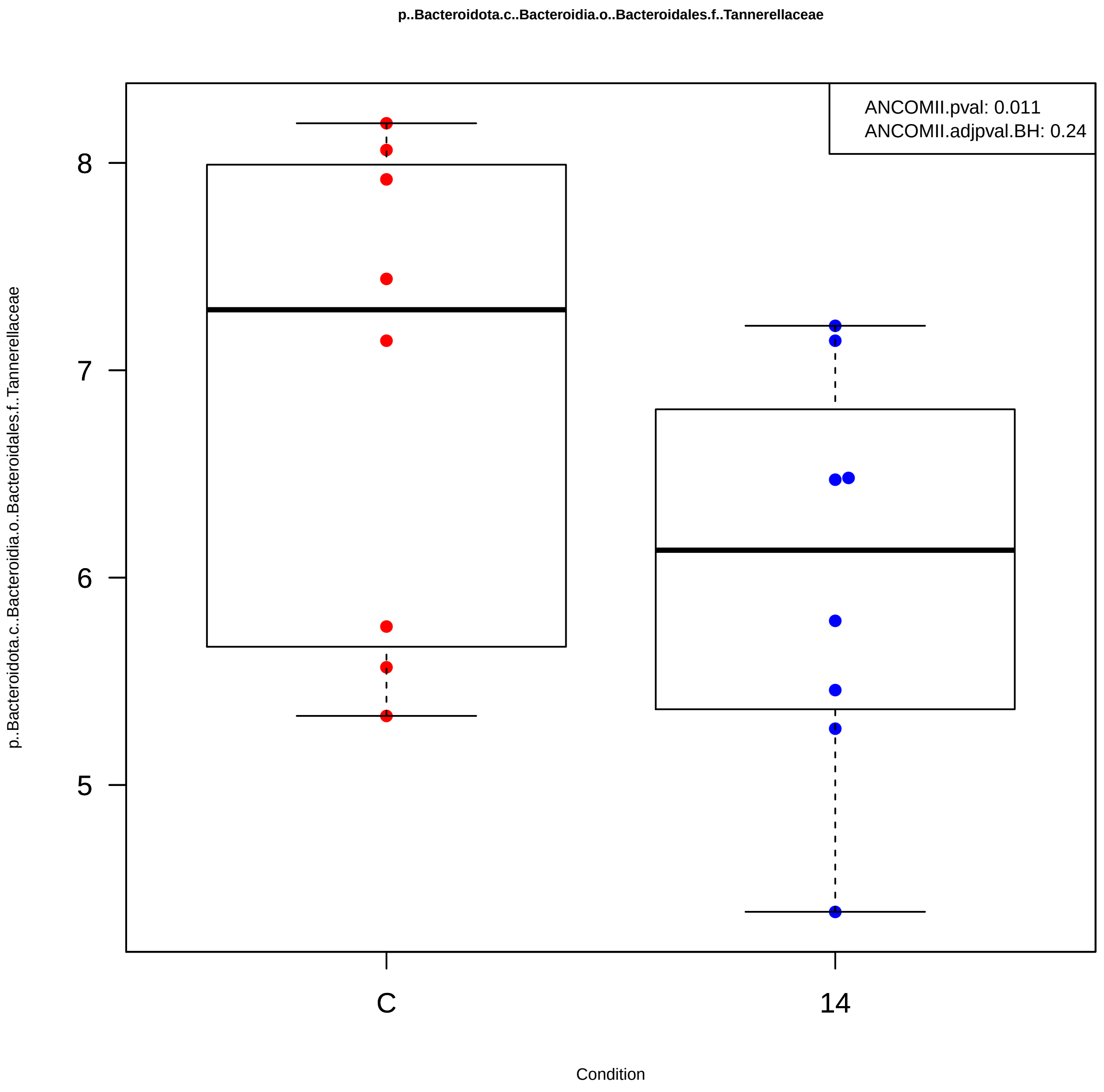
p..Bacteroidota.c..Bacteroidia.o..Bacteroidales.f..Tannerellaceae
ANCOMII.pval: 0.011
ANCOMII.adjpval.BH: 0.24
8
7
6
5
p..Bacteroidota.c..Bacteroidia.o..Bacteroidales.f..Tannerellaceae
C
14
Condition

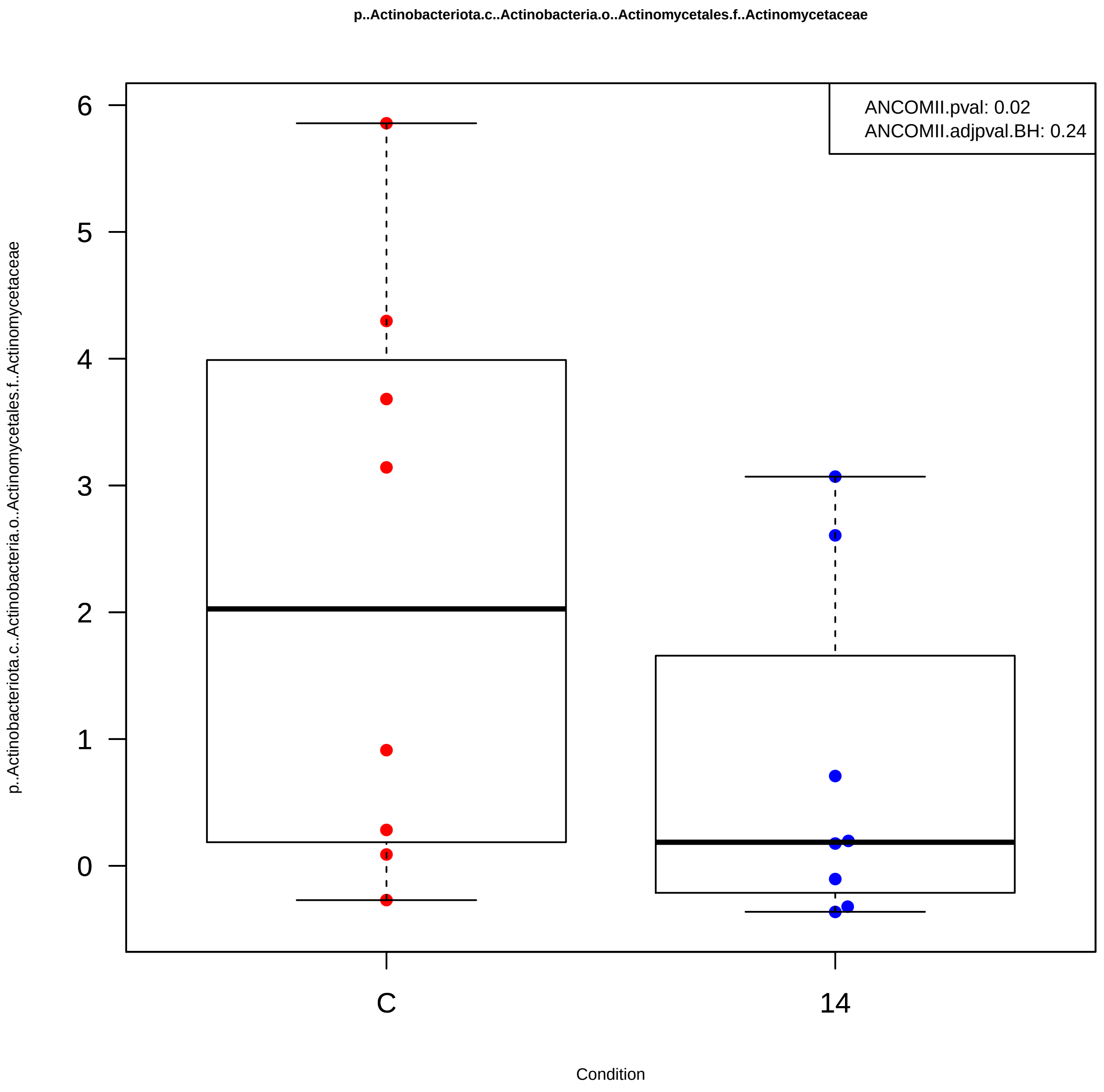

p..Actinobacteriota.c..Actinobacteria.o..Actinomycetales.f..Actinomycetaceae
ANCOMII.pval: 0.02
ANCOMII.adjpval.BH: 0.24
6
5
4
3
2
1
0
p..Actinobacteriota.c..Actinobacteria.o..Actinomycetales.f..Actinomycetaceae
C
14
Condition

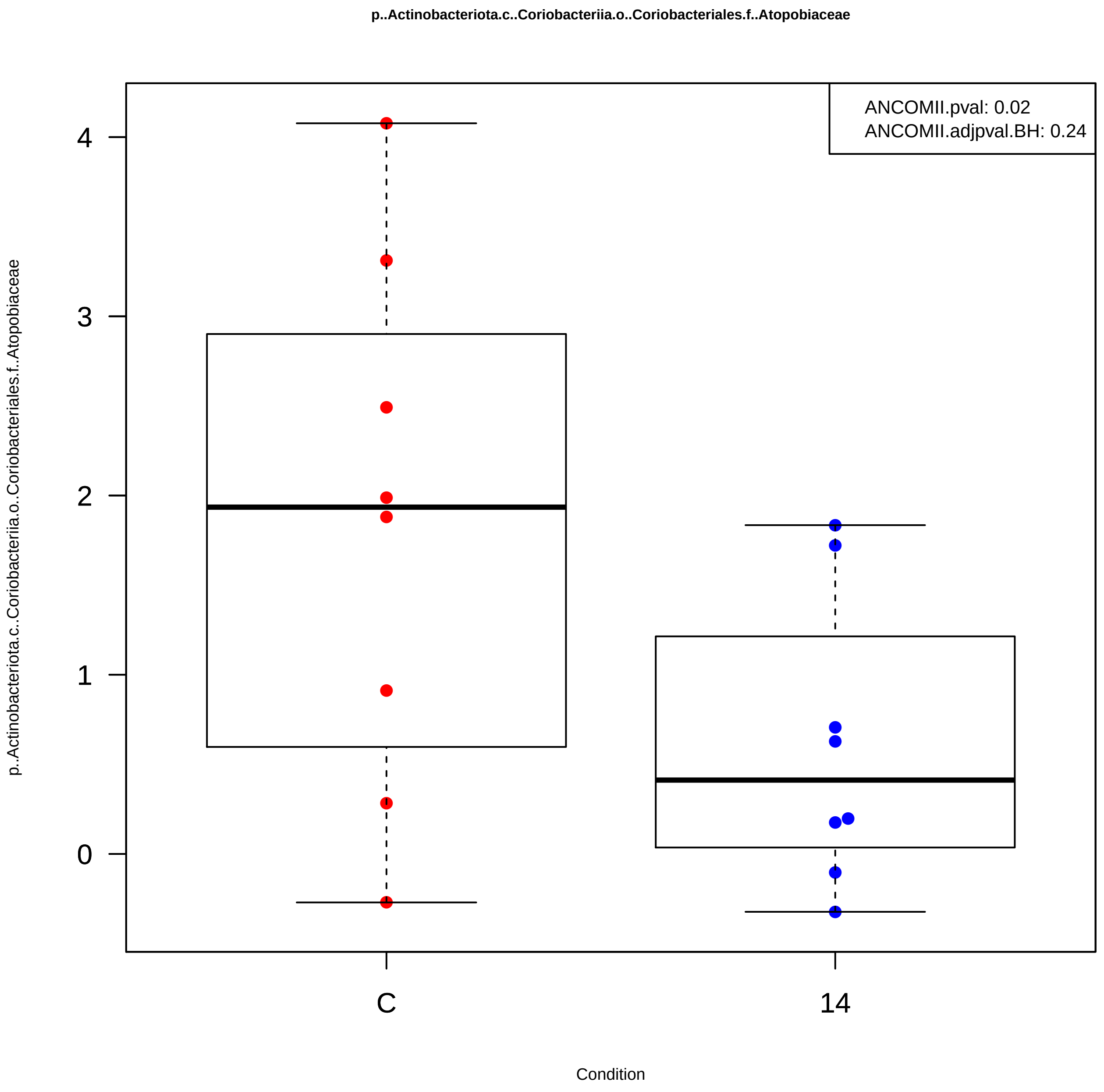

p..Actinobacteriota.c..Coriobacteriia.o..Coriobacteriales.f..Atopobiaceae
ANCOMII.pval: 0.02
ANCOMII.adjpval.BH: 0.24
p..Actinobacteriota.c..Coriobacteriia.o..Coriobacteriales.f..Atopobiaceae
4
3
2
1
0
C
14
Condition

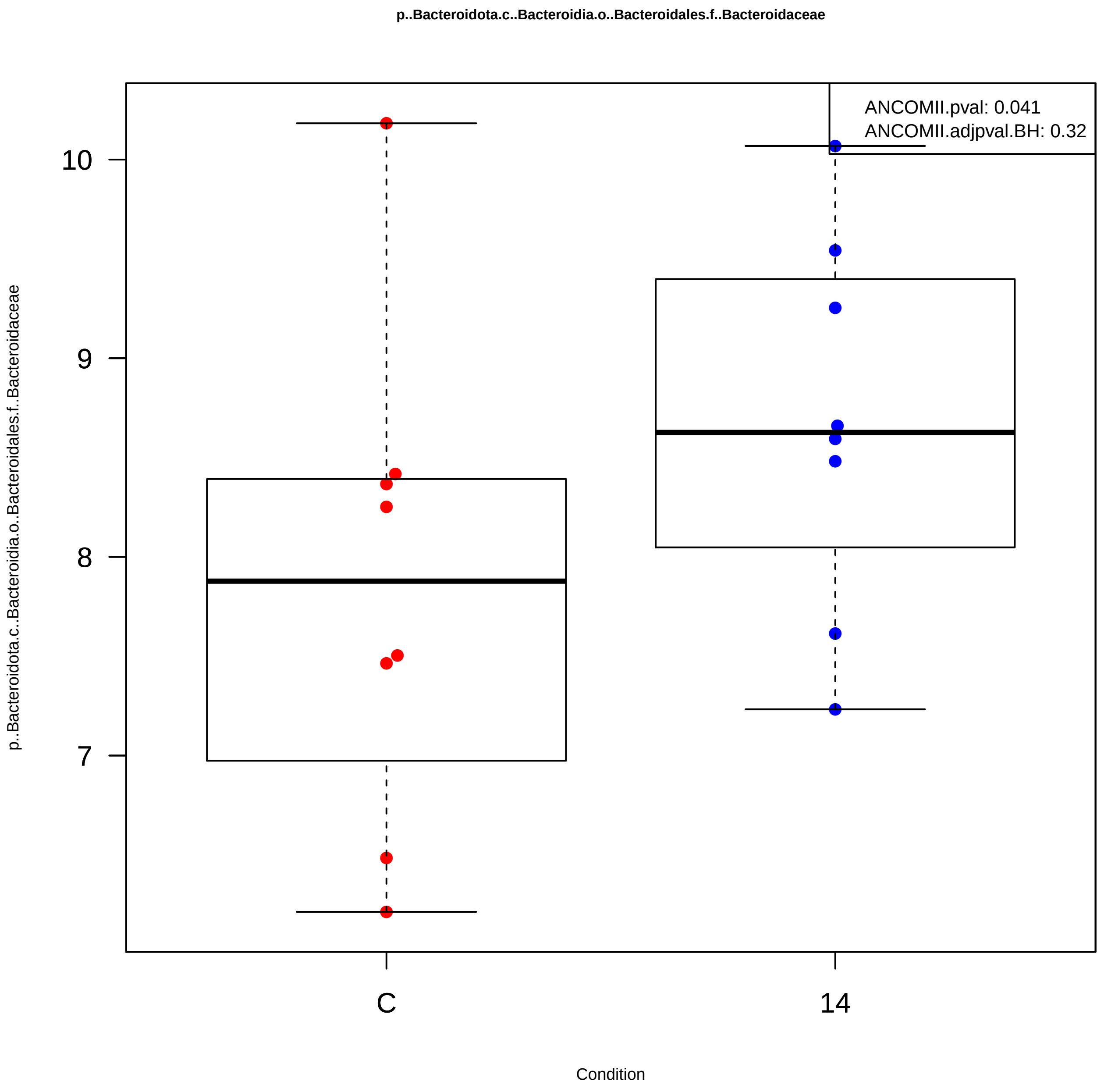

p..Bacteroidota.c..Bacteroidia.o..Bacteroidales.f..Bacteroidaceae
ANCOMII.pval: 0.041
ANCOMII.adjpval.BH: 0.32
10
9
8
7
p..Bacteroidota.c..Bacteroidia.o..Bacteroidales.f..Bacteroidaceae
C
14
Condition

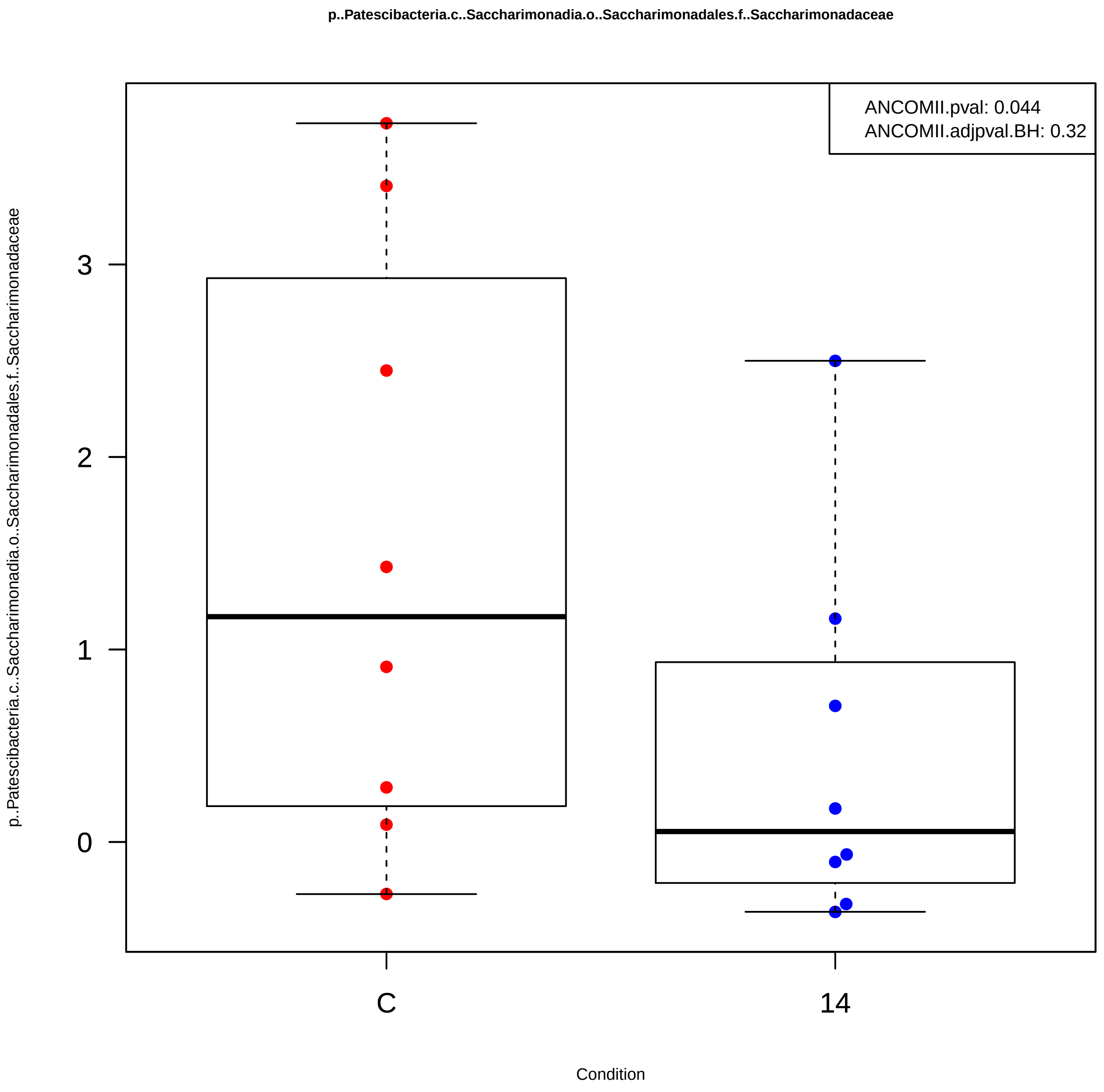
p..Patescibacteria.c..Saccharimonadia.o..Saccharimonadales.f..Saccharimonadaceae
ANCOMII.pval: 0.044
ANCOMII.adjpval.BH: 0.32
p..Patescibacteria.c..Saccharimonadia.o..Saccharimonadales.f..Saccharimonadaceae
0
1
2
3
C
14
Condition

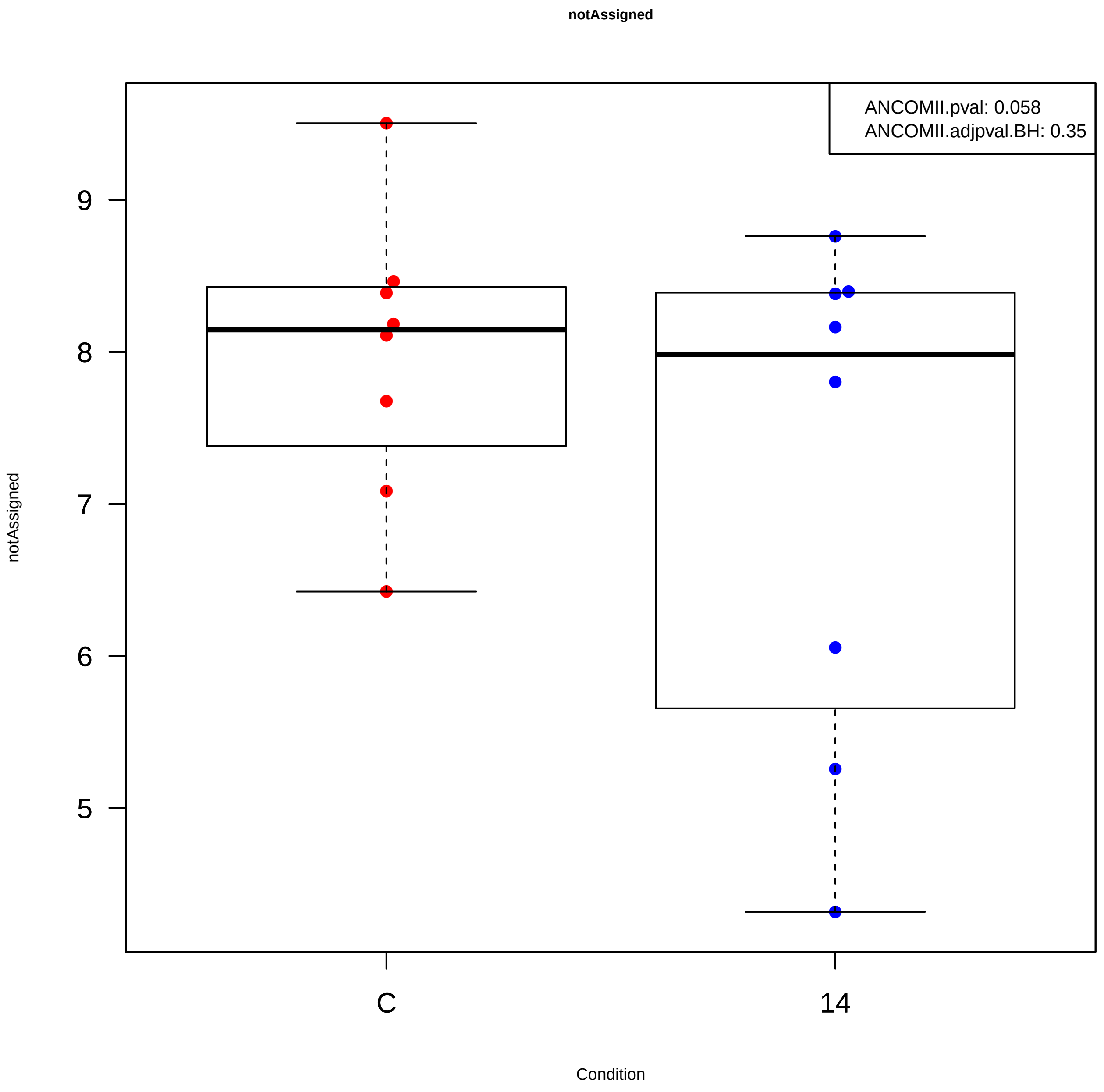
notAssigned
ANCOMII.pval: 0.058
ANCOMII.adjpval.BH: 0.35
9
8
7
6
5
notAssigned
C
14
Condition

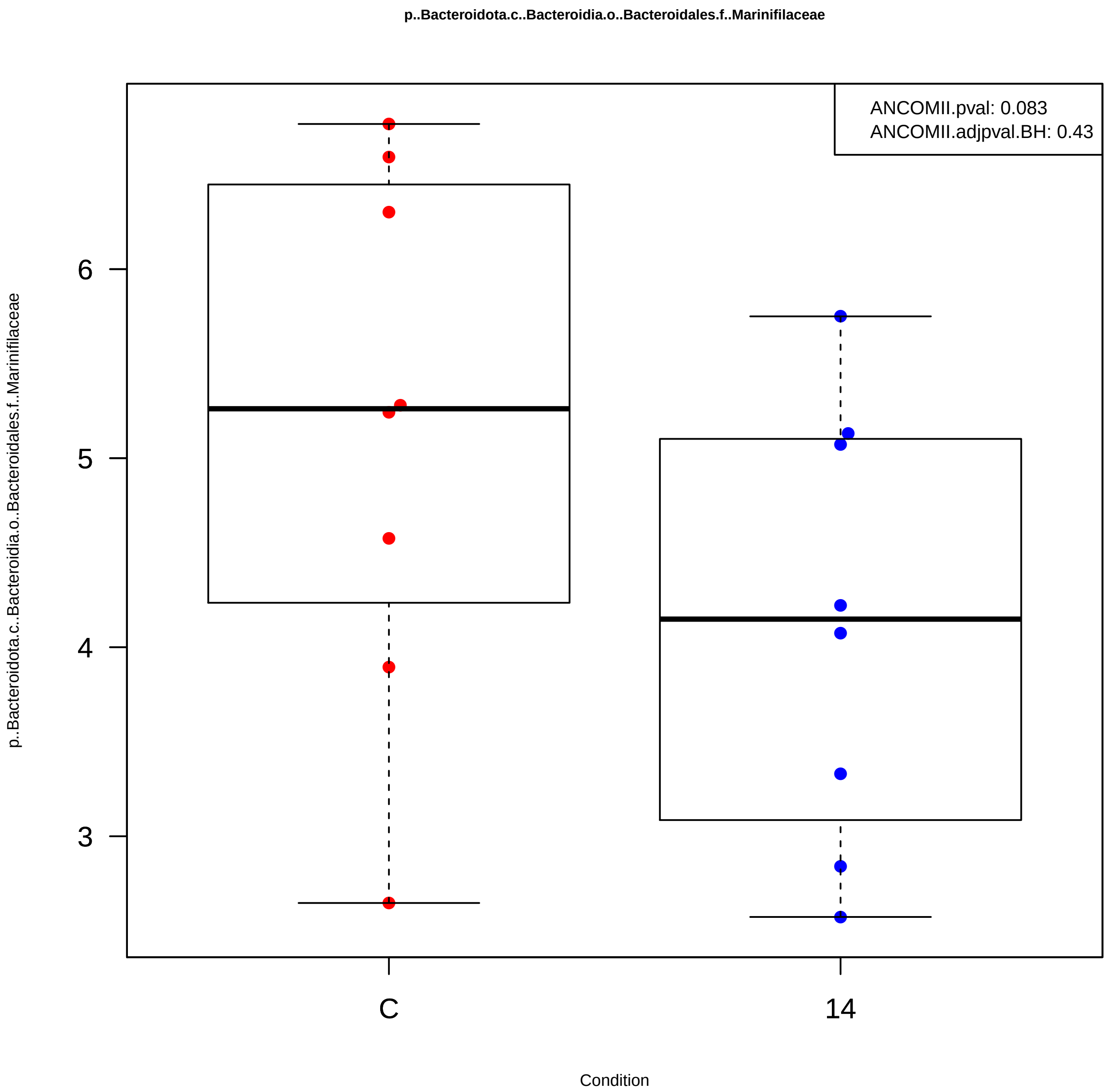
p..Bacteroidota.c..Bacteroidia.o..Bacteroidales.f..Marinifilaceae
ANCOMII.pval: 0.083
ANCOMII.adjpval.BH: 0.43
6
5
4
3
p..Bacteroidota.c..Bacteroidia.o..Bacteroidales.f..Marinifilaceae
C
14
Condition
