# Supplementary material for: Evaluation of the Effects of a Short Supplementation With Tannins on the Gut Microbiota of Healthy Subjects
Source: Front Microbiol. 2022 Apr 27;13:848611. doi: 10.3389/fmicb.2022.848611 (PMC9093706; doi:10.3389/fmicb.2022.848611)
Supplement: Supplementary file 2 [file Data_Sheet_2.PDF]

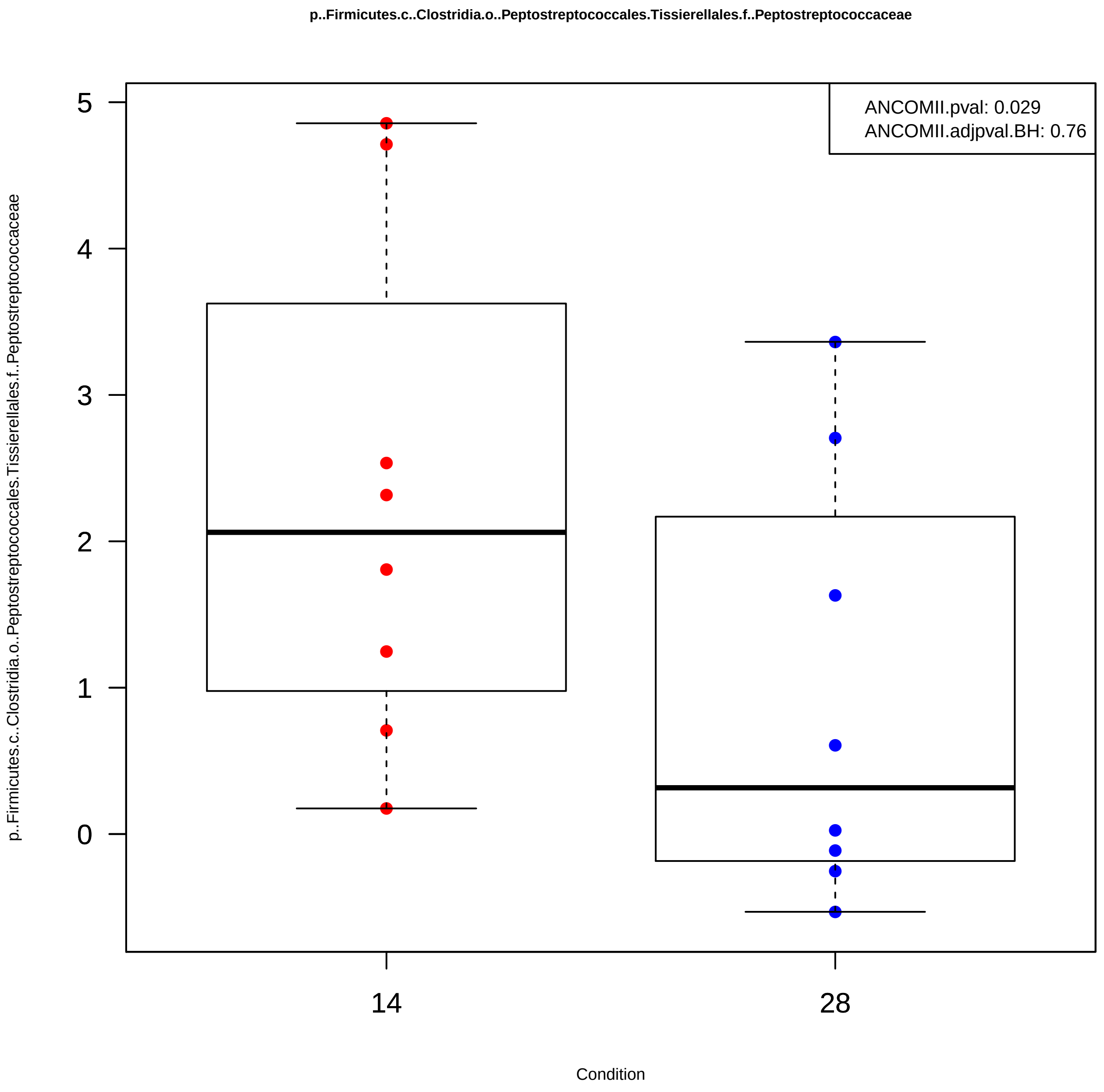

p..Firmicutes.c..Clostridia.o..Peptostreptococcales.Tissierellales.f..Peptostreptococcaceae
ANCOMII.pval: 0.029
ANCOMII.adjpval.BH: 0.76
p..Firmicutes.c..Clostridia.o..Peptostreptococcales.Tissierellales.f..Peptostreptococcaceae
0
1
2
3
4
5
14
28
Condition

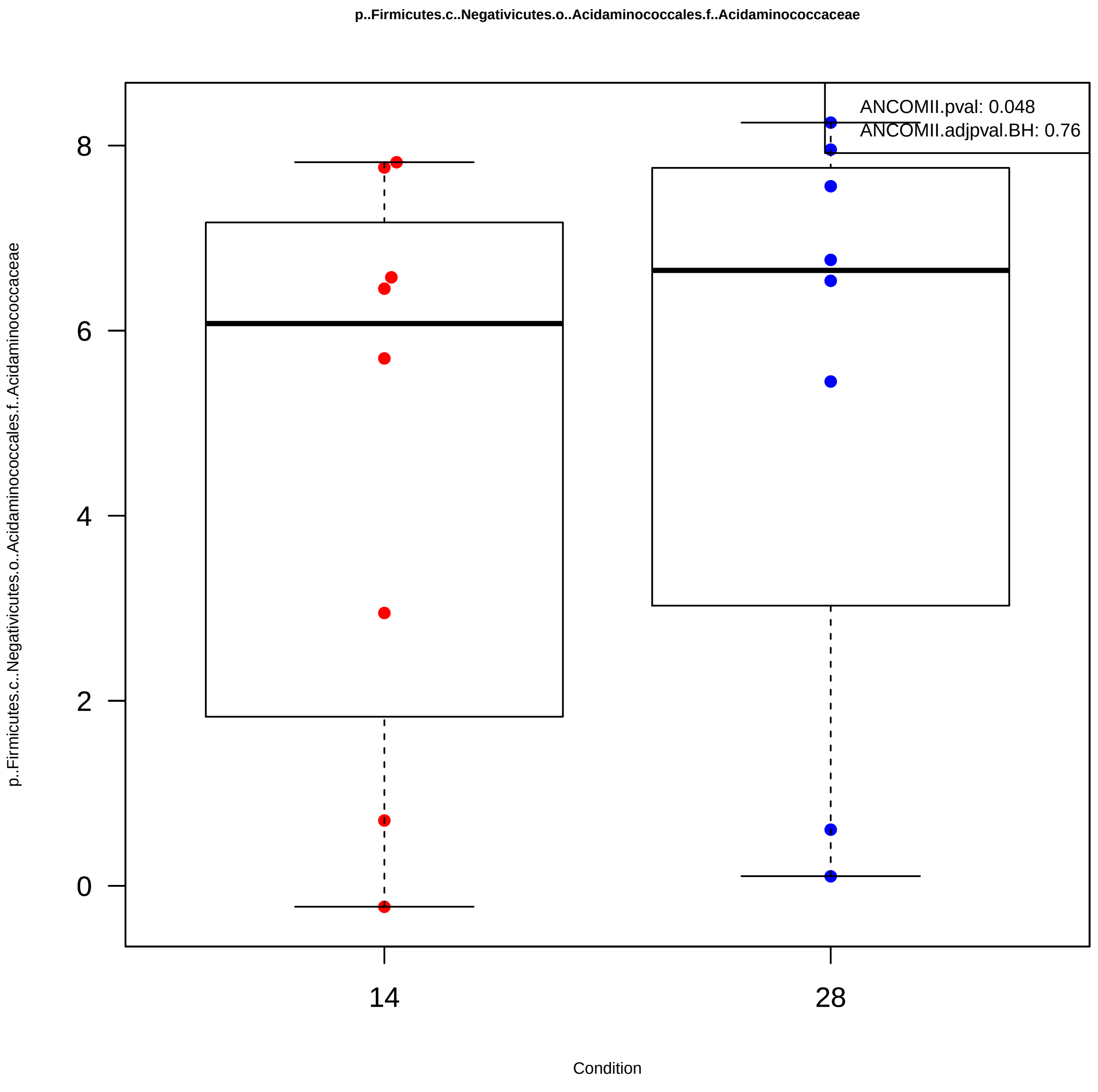
p..Firmicutes.c..Negativicutes.o..Acidaminococcales.f..Acidaminococcaceae
ANCOMII.pval: 0.048
ANCOMII.adjpval.BH: 0.76
8
6
4
2
0
14
28
Condition
p..Firmicutes.c..Negativicutes.o..Acidaminococcales.f..Acidaminococcaceae

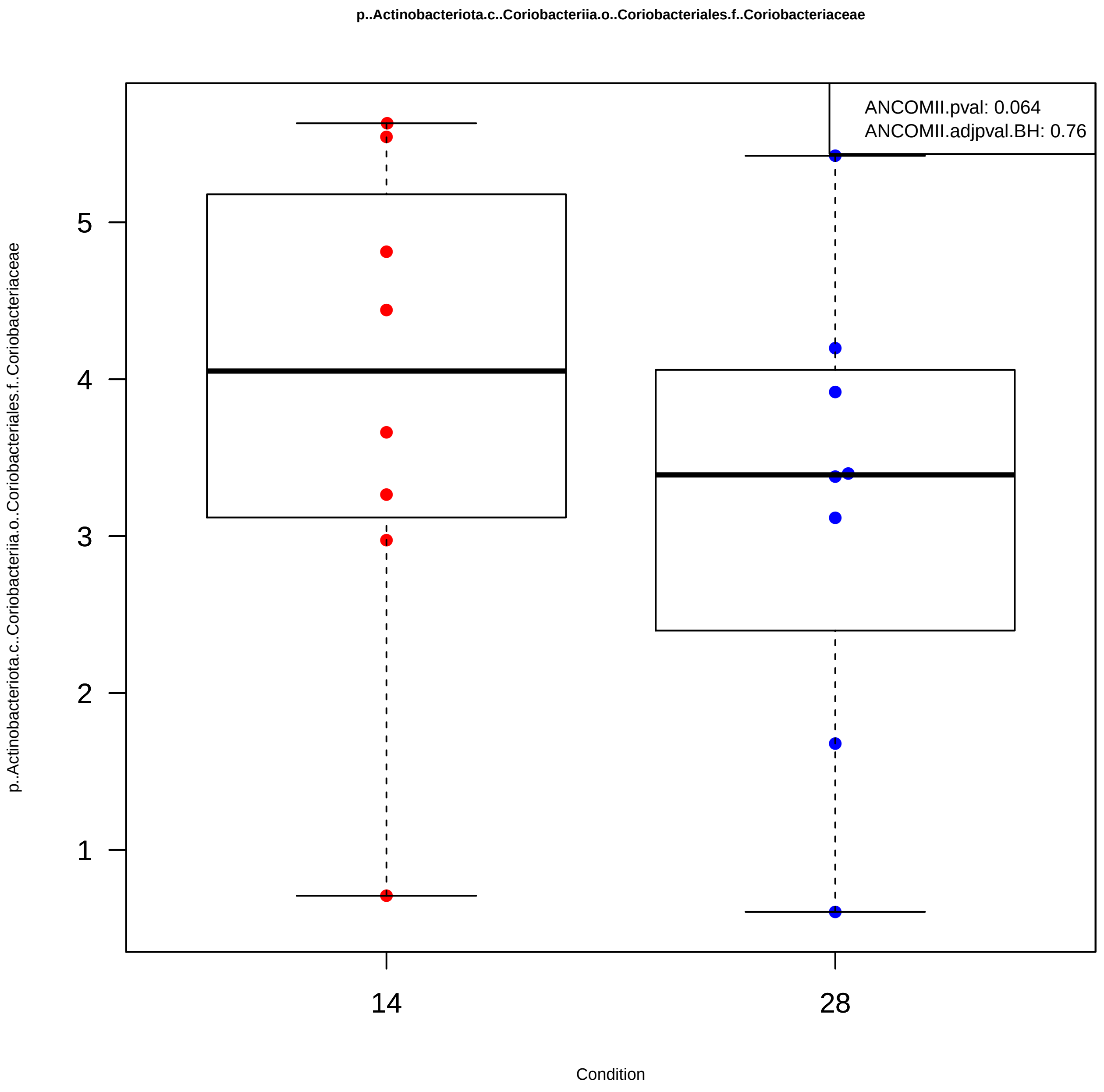
p..Actinobacteriota.c..Coriobacteriia.o..Coriobacteriales.f..Coriobacteriaceae
ANCOMII.pval: 0.064
ANCOMII.adjpval.BH: 0.76
p..Actinobacteriota.c..Coriobacteriia.o..Coriobacteriales.f..Coriobacteriaceae
1
2
3
4
5
14
28
Condition
