# Supplementary material for: Evaluation of the Effects of a Short Supplementation With Tannins on the Gut Microbiota of Healthy Subjects
Source: Front Microbiol. 2022 Apr 27;13:848611. doi: 10.3389/fmicb.2022.848611 (PMC9093706; doi:10.3389/fmicb.2022.848611)
Supplement: Supplementary file 3 [file Data_Sheet_3.PDF]

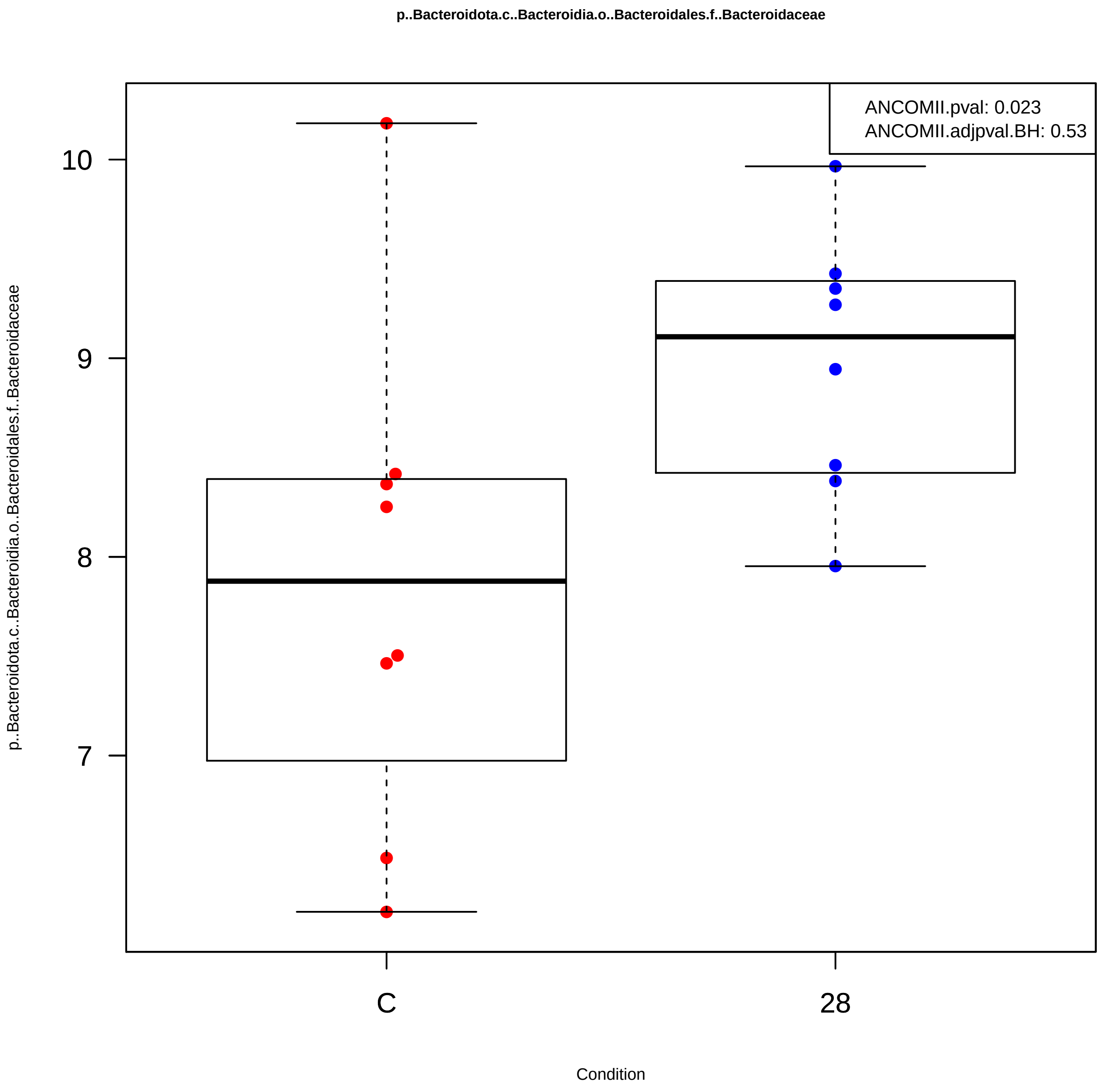
p..Bacteroidota.c..Bacteroidia.o..Bacteroidales.f..Bacteroidaceae
ANCOMII.pval: 0.023
ANCOMII.adjpval.BH: 0.53
10
9
8
7
p..Bacteroidota.c..Bacteroidia.o..Bacteroidales.f..Bacteroidaceae
C
28
Condition

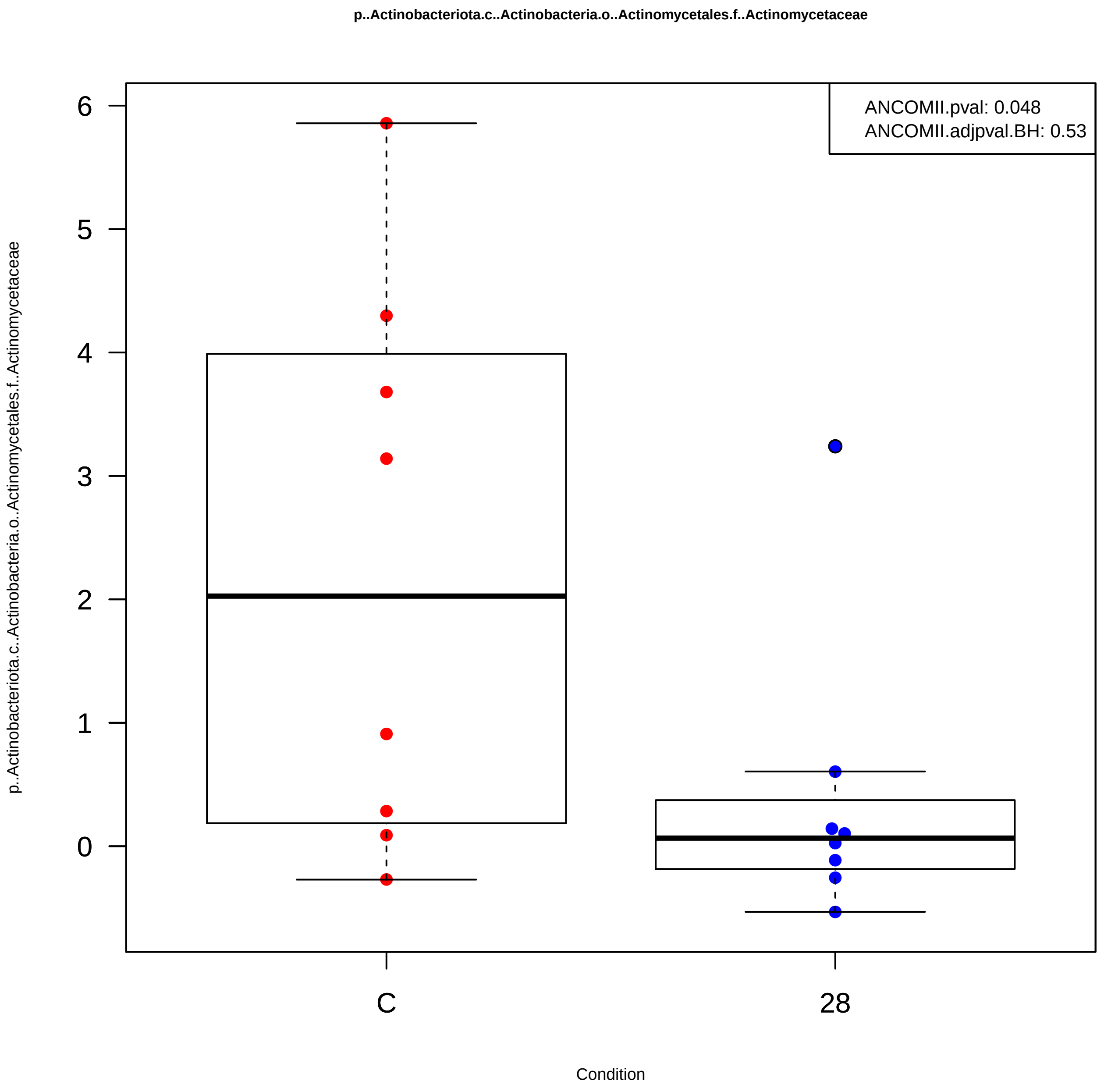

p..Actinobacteriota.c..Actinobacteria.o..Actinomycetales.f..Actinomycetaceae
ANCOMII.pval: 0.048
ANCOMII.adjpval.BH: 0.53
6
5
4
3
2
1
0
C
28
Condition
p..Actinobacteriota.c..Actinobacteria.o..Actinomycetales.f..Actinomycetaceae

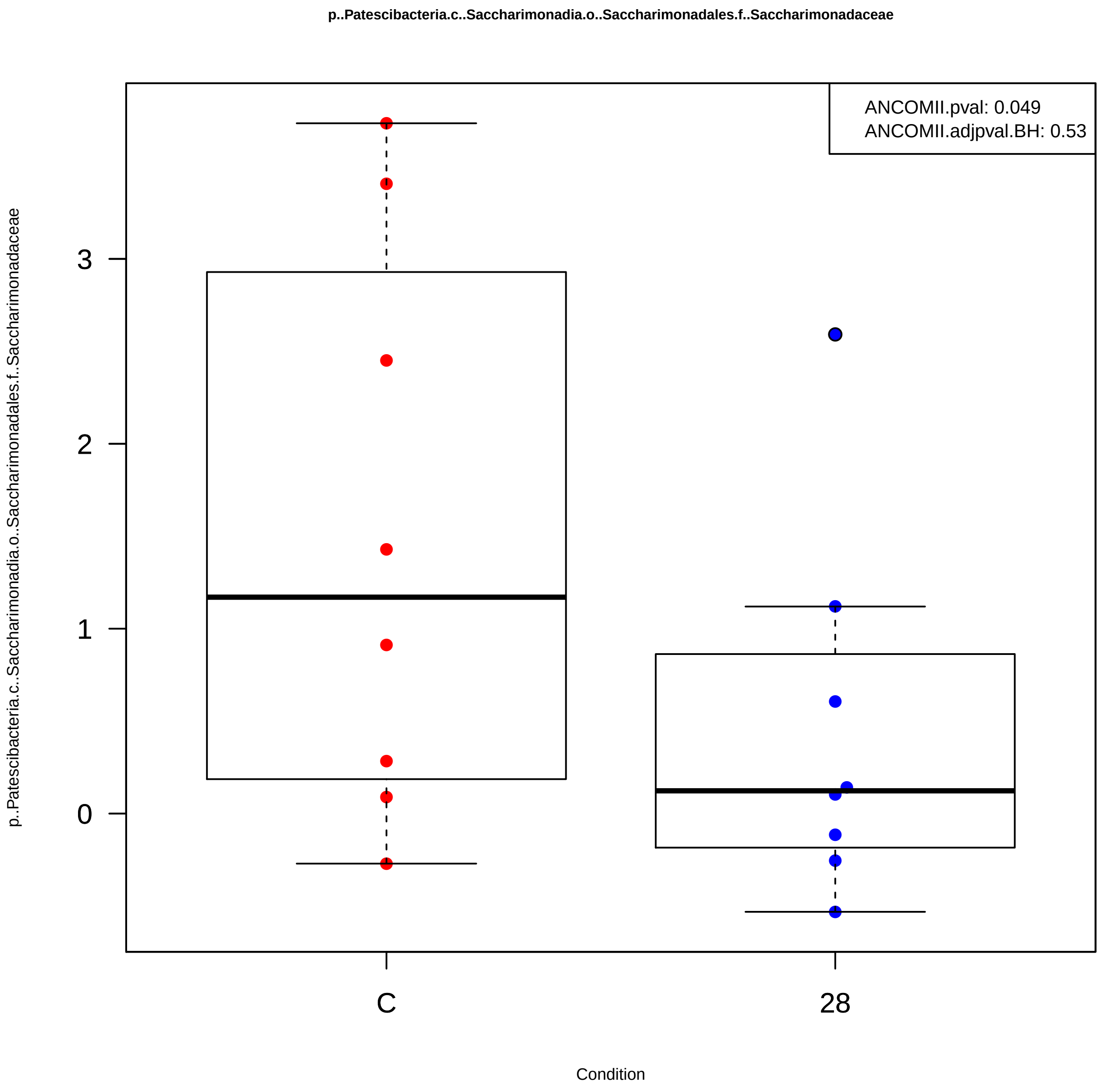

p..Patescibacteria.c..Saccharimonadia.o..Saccharimonadales.f..Saccharimonadaceae
ANCOMII.pval: 0.049
ANCOMII.adjpval.BH: 0.53
p..Patescibacteria.c..Saccharimonadia.o..Saccharimonadales.f..Saccharimonadaceae
0
1
2
3
C
28
Condition

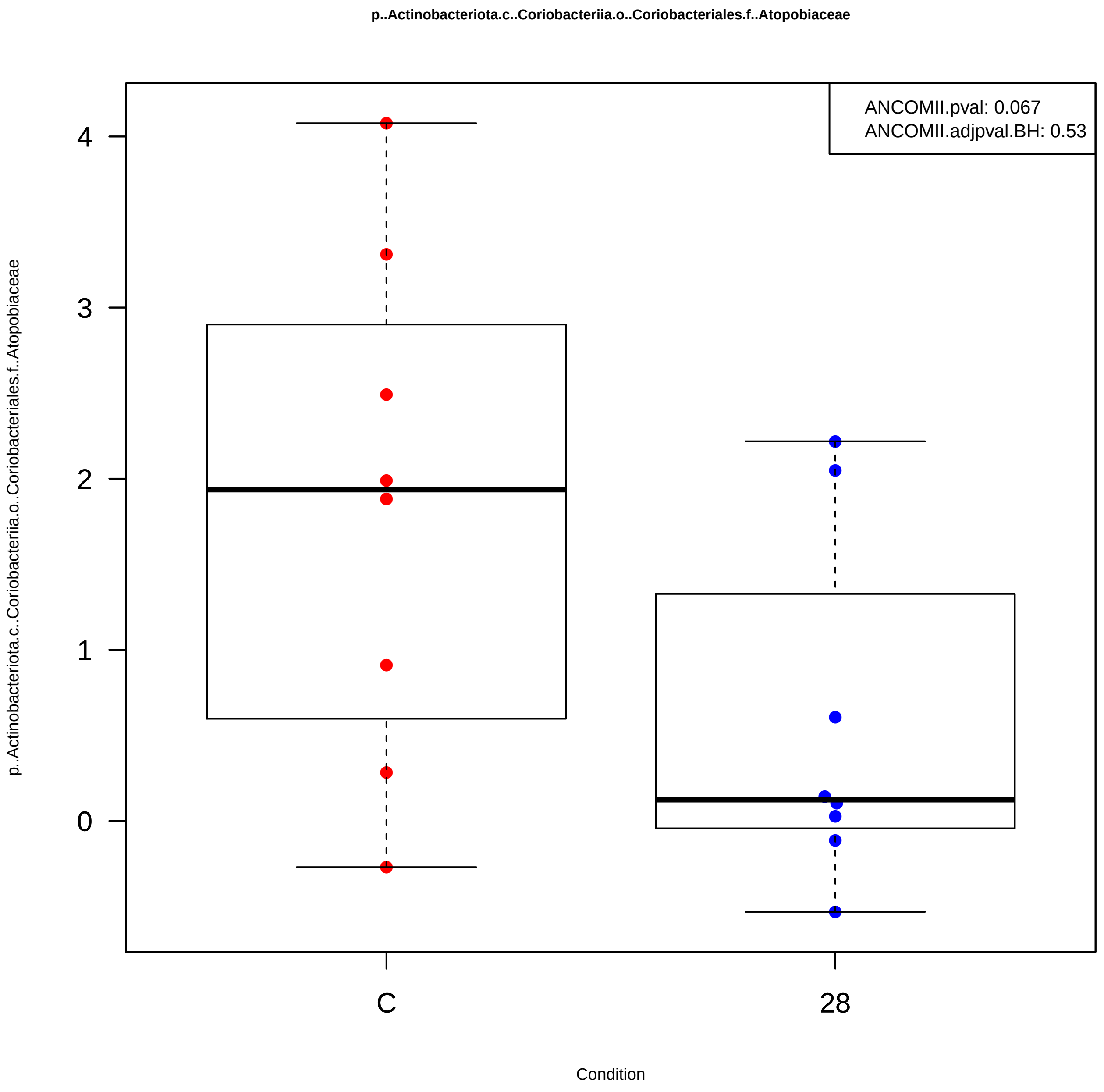

p..Actinobacteriota.c..Coriobacteriia.o..Coriobacteriales.f..Atopobiaceae
ANCOMII.pval: 0.067
ANCOMII.adjpval.BH: 0.53
4
3
2
1
0
p..Actinobacteriota.c..Coriobacteriia.o..Coriobacteriales.f..Atopobiaceae
C
28
Condition

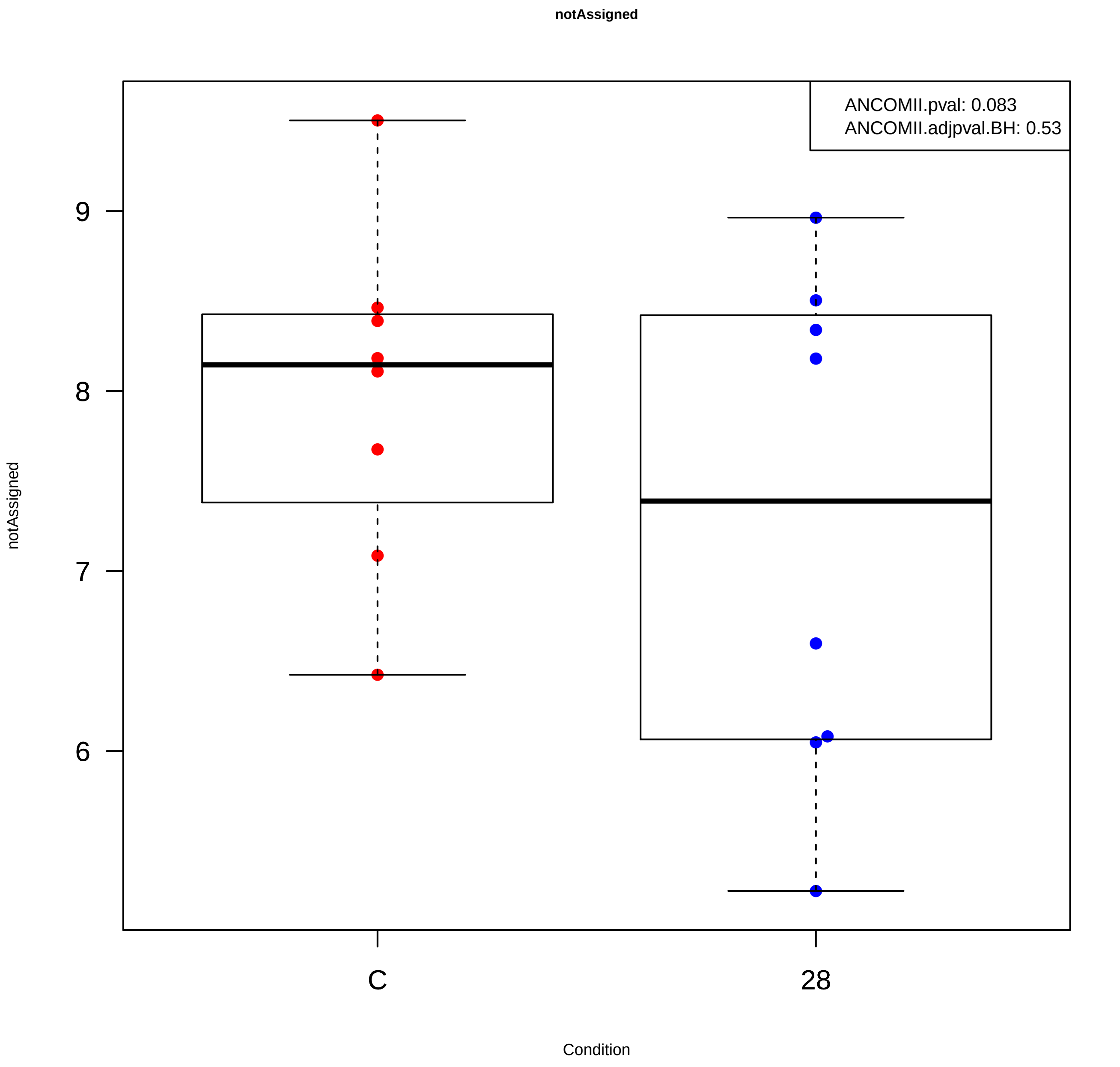
notAssigned
ANCOMII.pval: 0.083
ANCOMII.adjpval.BH: 0.53
9
8
7
6
notAssigned
C
28
Condition

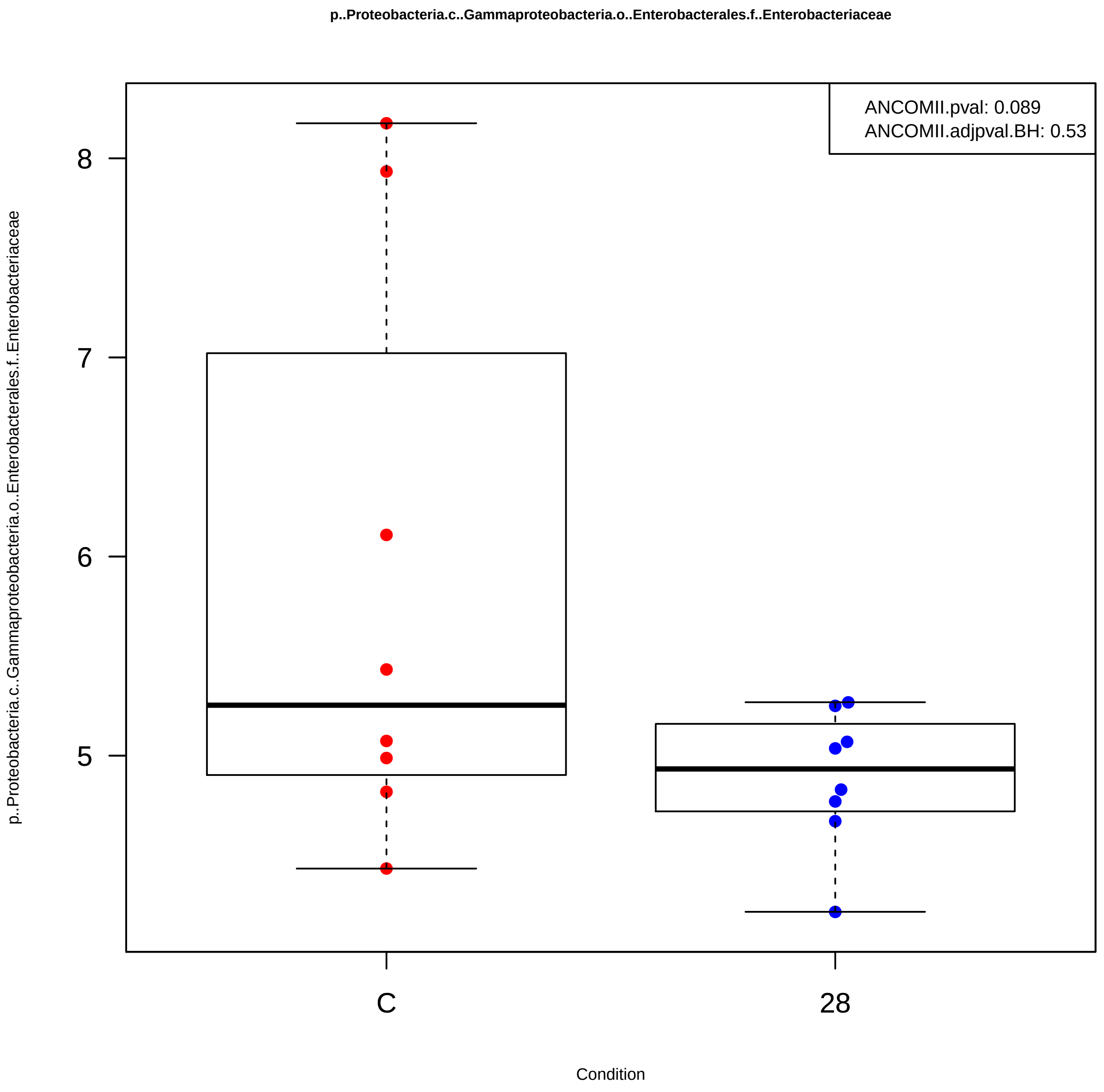
p..Proteobacteria.c..Gammaproteobacteria.o..Enterobacterales.f..Enterobacteriaceae
ANCOMII.pval: 0.089
ANCOMII.adjpval.BH: 0.53
8
7
6
5
p..Proteobacteria.c..Gammaproteobacteria.o..Enterobacterales.f..Enterobacteriaceae
C
28
Condition
