# Supplementary material for: Evaluation of the Effects of a Short Supplementation With Tannins on the Gut Microbiota of Healthy Subjects
Source: Front Microbiol. 2022 Apr 27;13:848611. doi: 10.3389/fmicb.2022.848611 (PMC9093706; doi:10.3389/fmicb.2022.848611)
Supplement: Supplementary file 4 [file Data_Sheet_4.PDF]

p..Firmicutes.c..Bacilli.o..Lactobacillales.f..Streptococcaceae.g..Streptococcus.s..cristatus

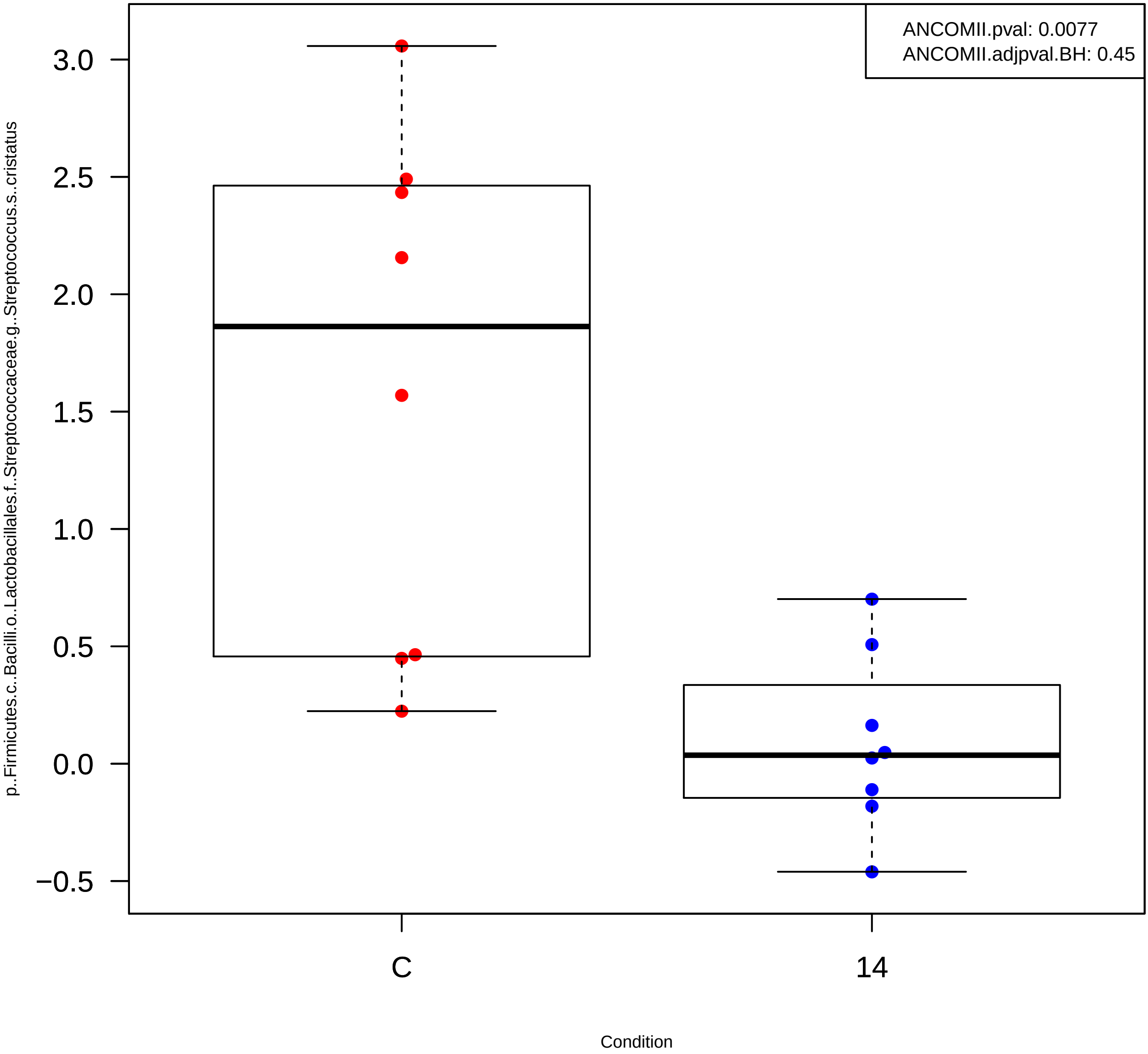

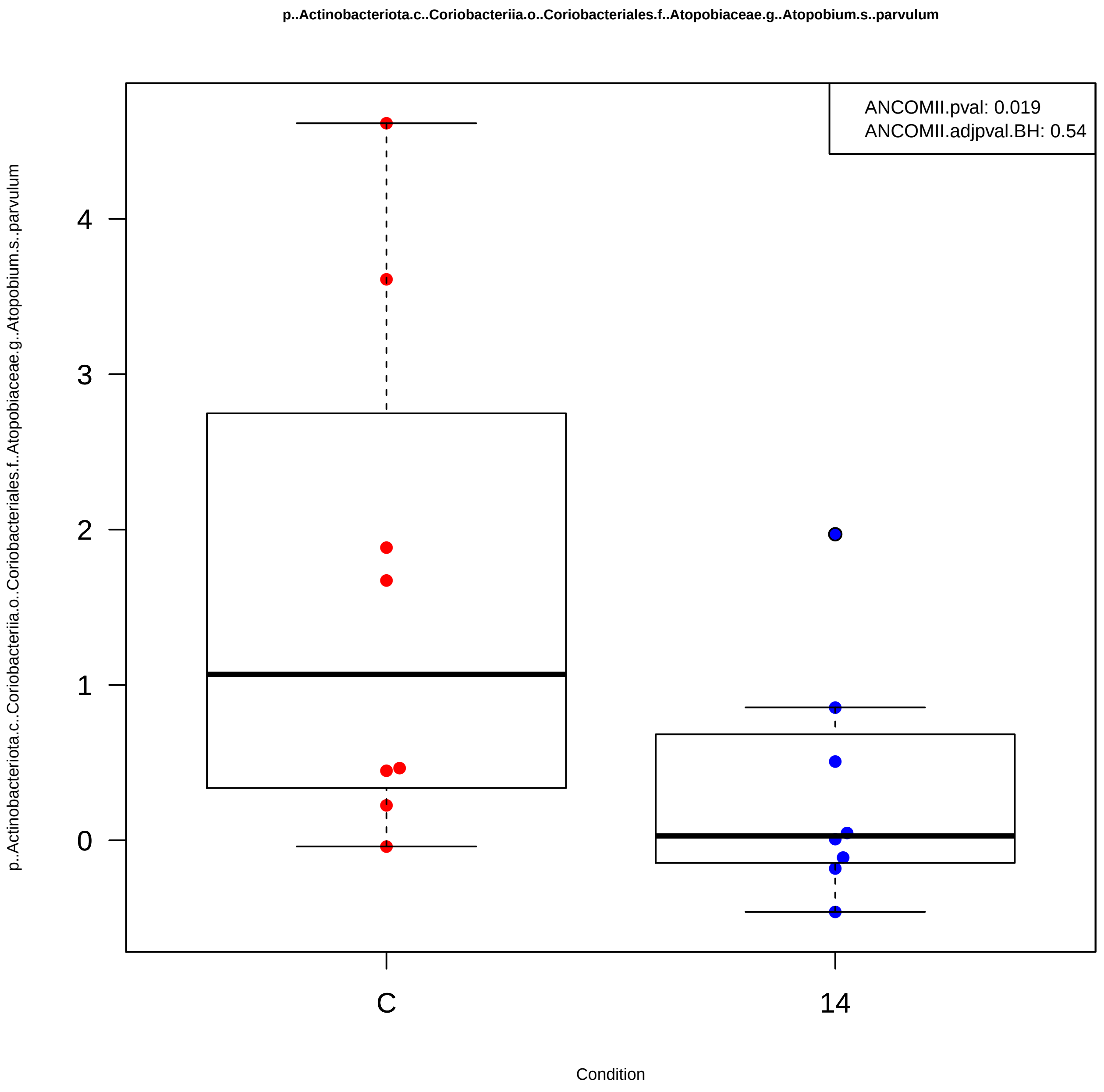

p..Actinobacteriota.c..Coriobacteriia.o..Coriobacteriales.f..Atopobiaceae.g..Atopobium.s..parvulum
ANCOMII.pval: 0.019
ANCOMII.adjpval.BH: 0.54
p..Actinobacteriota.c..Coriobacteriia.o..Coriobacteriales.f..Atopobiaceae.g..Atopobium.s..parvulum
0
1
2
3
4
C
14
Condition

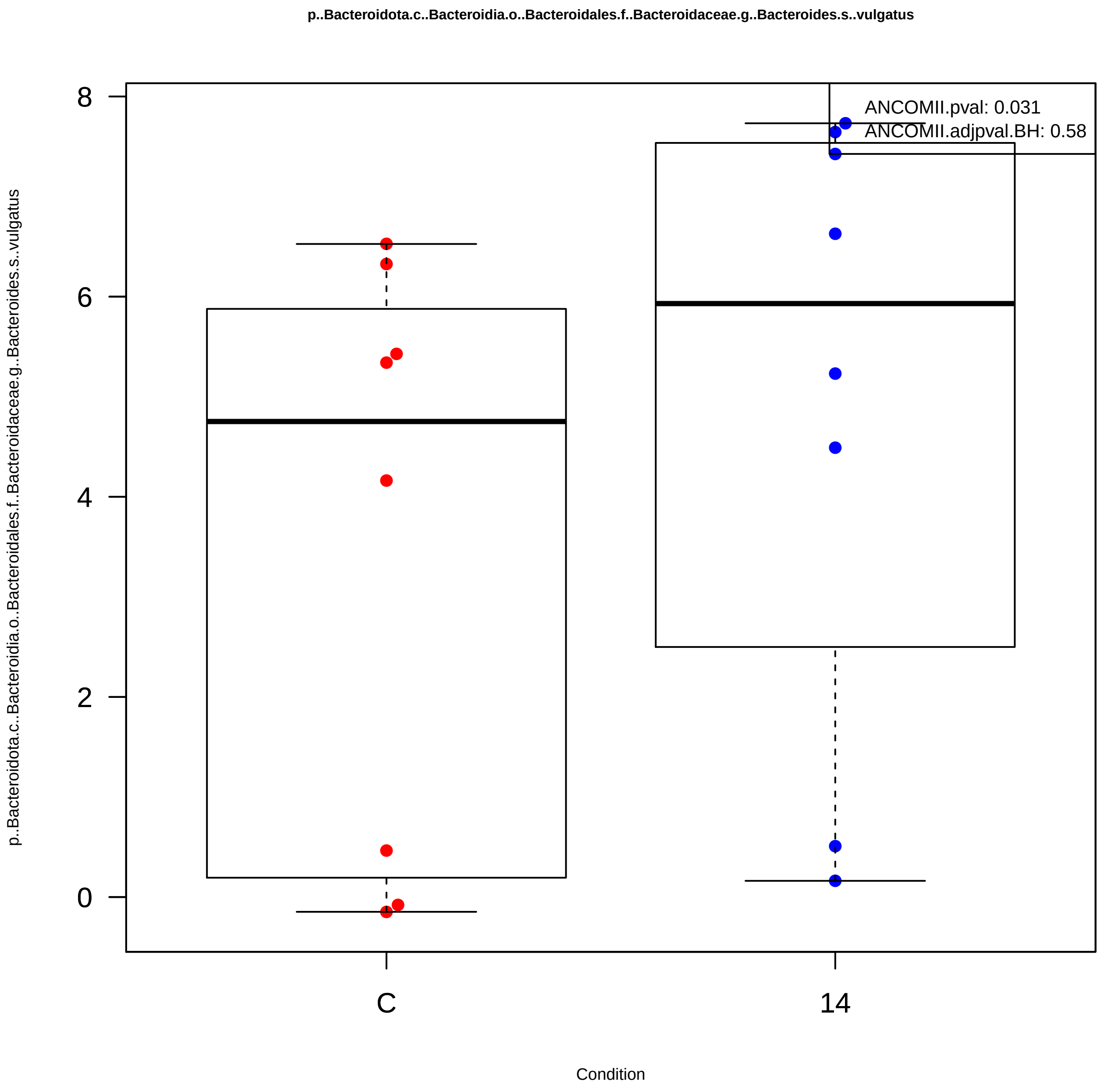
p..Bacteroidota.c..Bacteroidia.o..Bacteroidales.f..Bacteroidaceae.g..Bacteroides.s..vulgatus
ANCOMII.pval: 0.031
ANCOMII.adjpval.BH: 0.58
8
6
4
2
0
C
14
Condition
p..Bacteroidota.c..Bacteroidia.o..Bacteroidales.f..Bacteroidaceae.g..Bacteroides.s..vulgatus

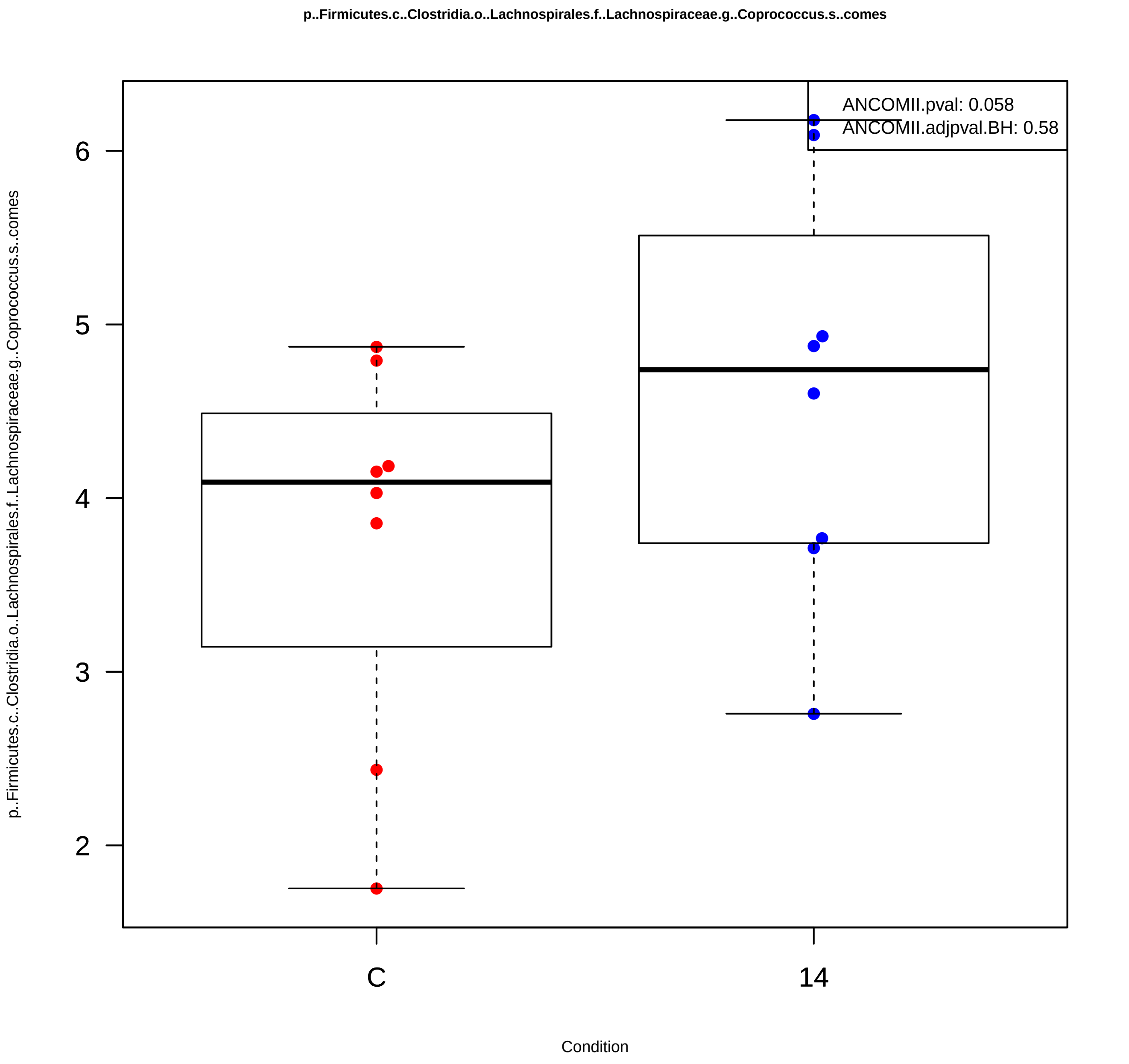

p..Firmicutes.c..Clostridia.o..Lachnospirales.f..Lachnospiraceae.g..Coprococcus.s..comes
ANCOMII.pval: 0.058
ANCOMII.adjpval.BH: 0.58
6
5
4
3
2
C
14
Condition
p..Firmicutes.c..Clostridia.o..Lachnospirales.f..Lachnospiraceae.g..Coprococcus.s..comes

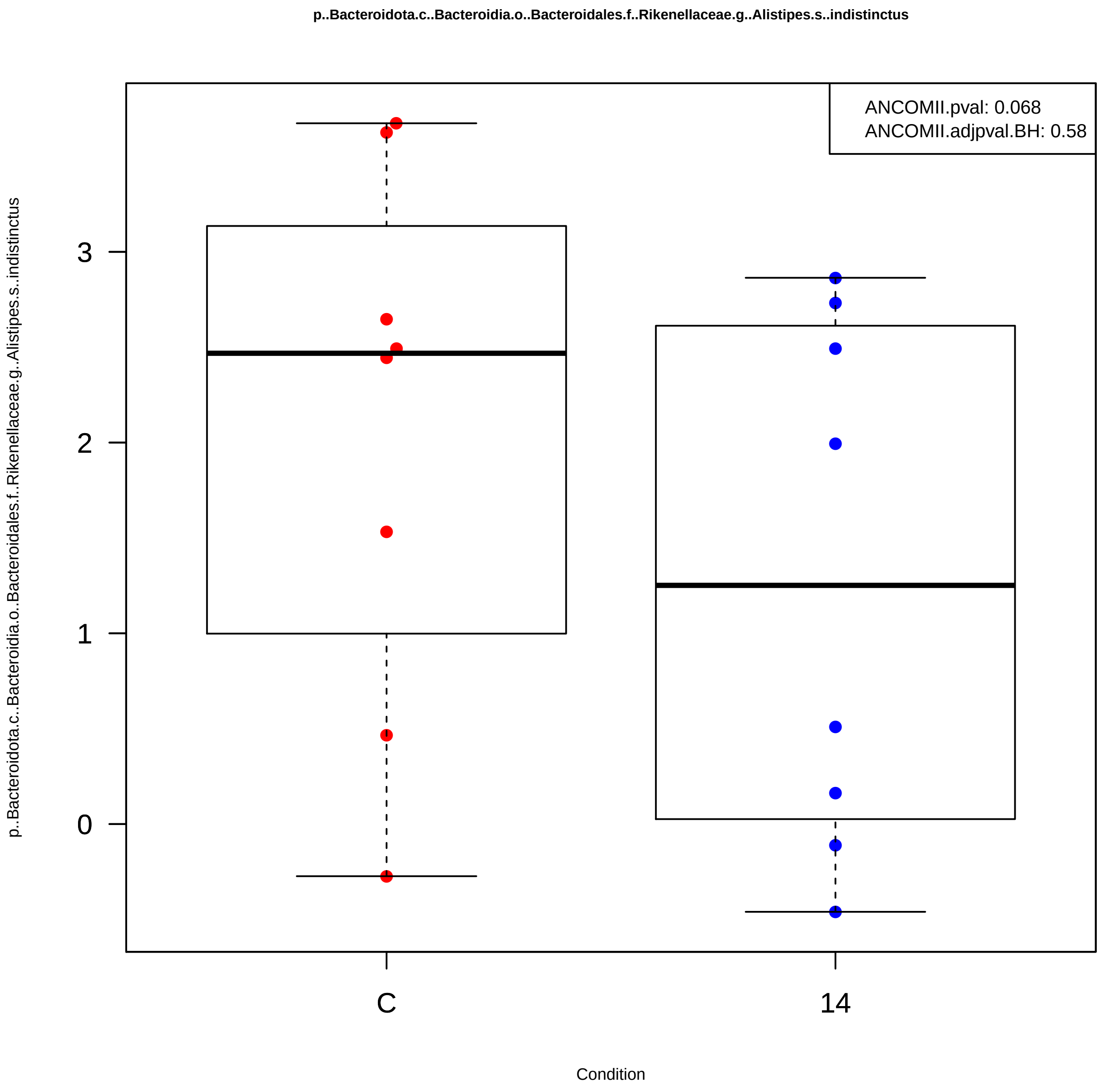
p..Bacteroidota.c..Bacteroidia.o..Bacteroidales.f..Rikenellaceae.g..Alistipes.s..indistinctus
ANCOMII.pval: 0.068
ANCOMII.adjpval.BH: 0.58
p..Bacteroidota.c..Bacteroidia.o..Bacteroidales.f..Rikenellaceae.g..Alistipes.s..indistinctus
0
1
2
3
C
14
Condition

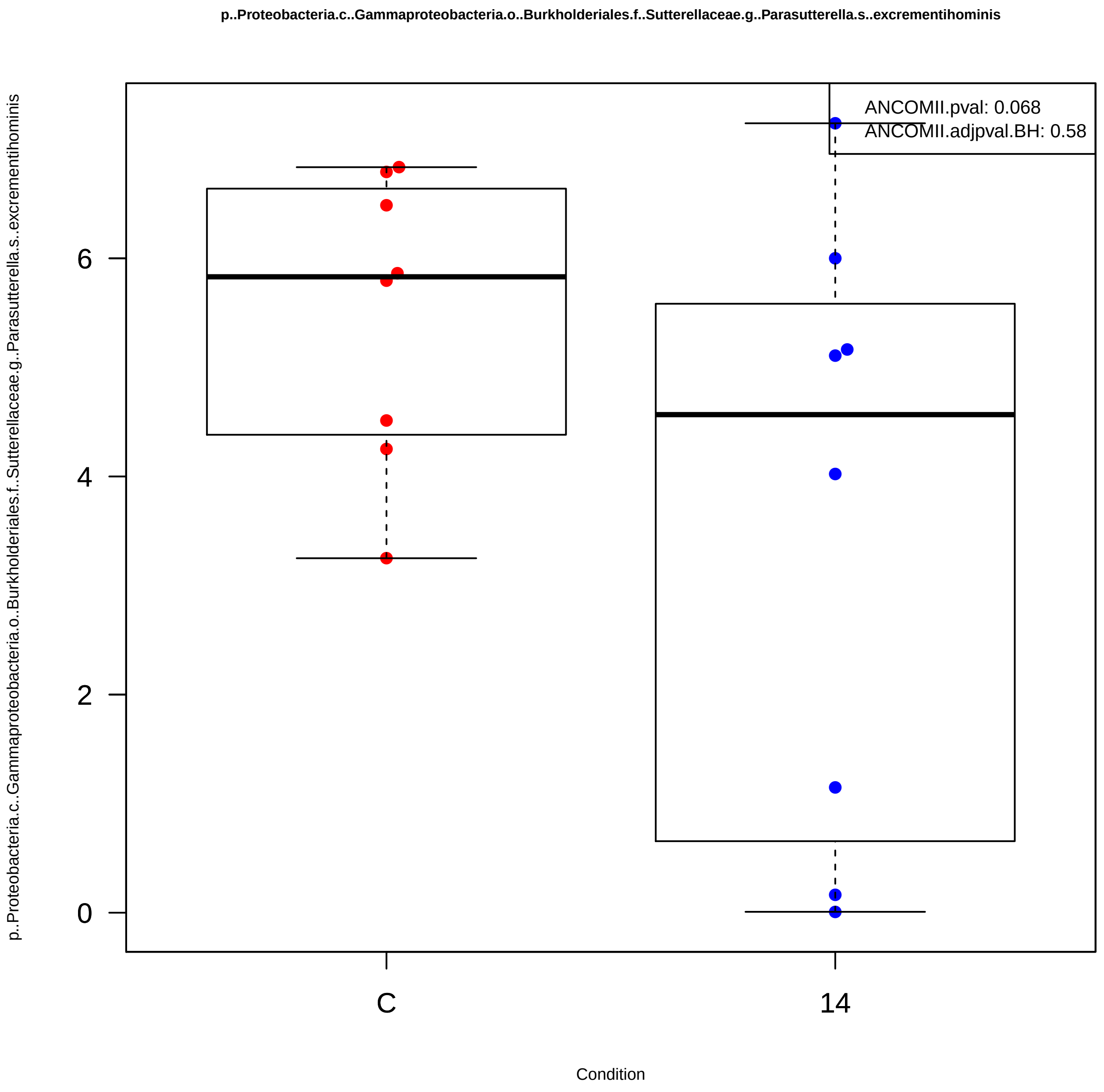
p..Proteobacteria.c..Gammaproteobacteria.o..Burkholderiales.f..Sutterellaceae.g..Parasutterella.s..excrementihominis
ANCOMII.pval: 0.068
ANCOMII.adjpval.BH: 0.58
p..Proteobacteria.c..Gammaproteobacteria.o..Burkholderiales.f..Sutterellaceae.g..Parasutterella.s..excrementihominis
0
2
4
6
C
14
Condition

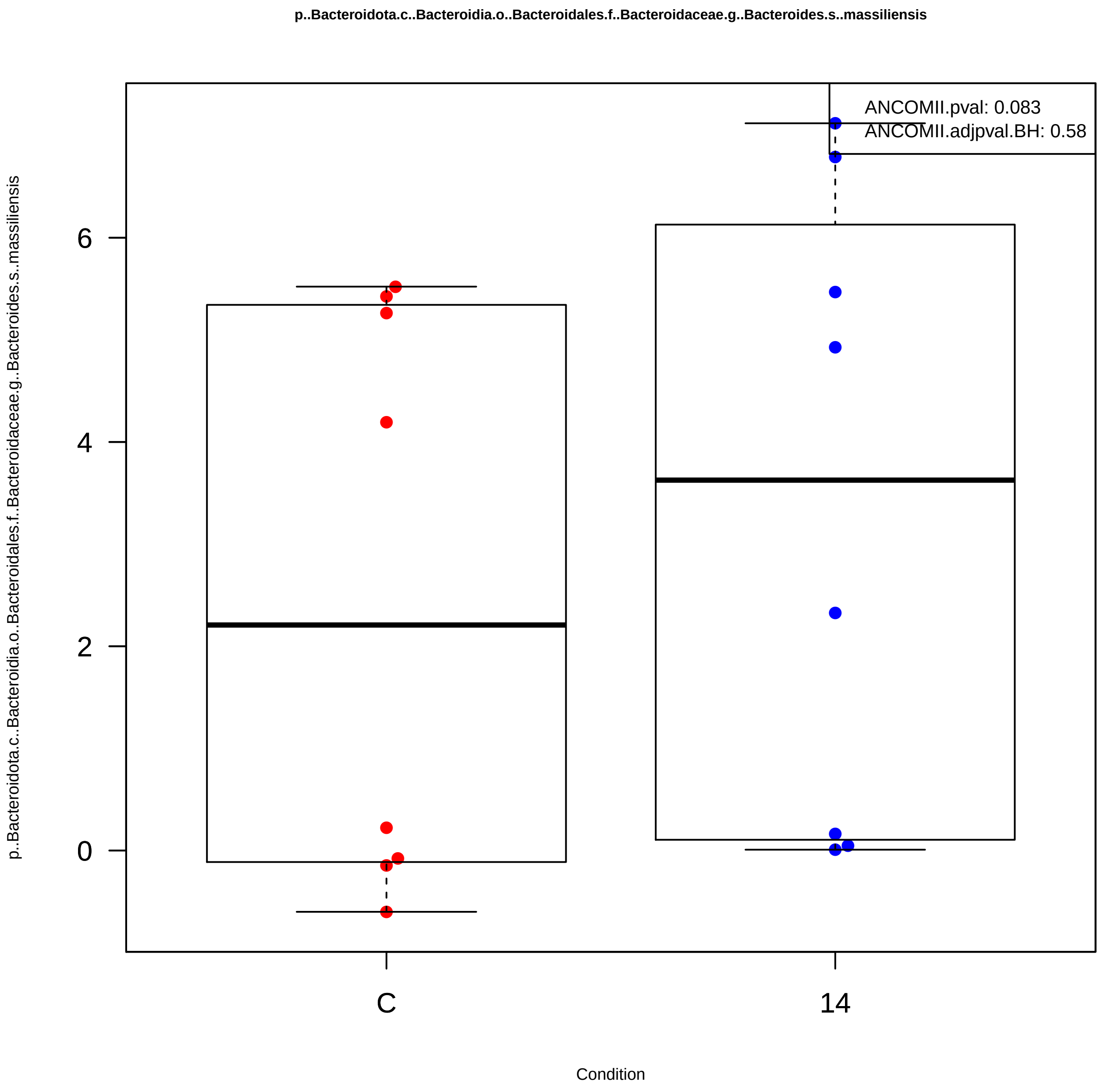

p..Bacteroidota.c..Bacteroidia.o..Bacteroidales.f..Bacteroidaceae.g..Bacteroides.s..massiliensis
ANCOMII.pval: 0.083
ANCOMII.adjpval.BH: 0.58
6
4
2
0
p..Bacteroidota.c..Bacteroidia.o..Bacteroidales.f..Bacteroidaceae.g..Bacteroides.s..massiliensis
C
14
Condition

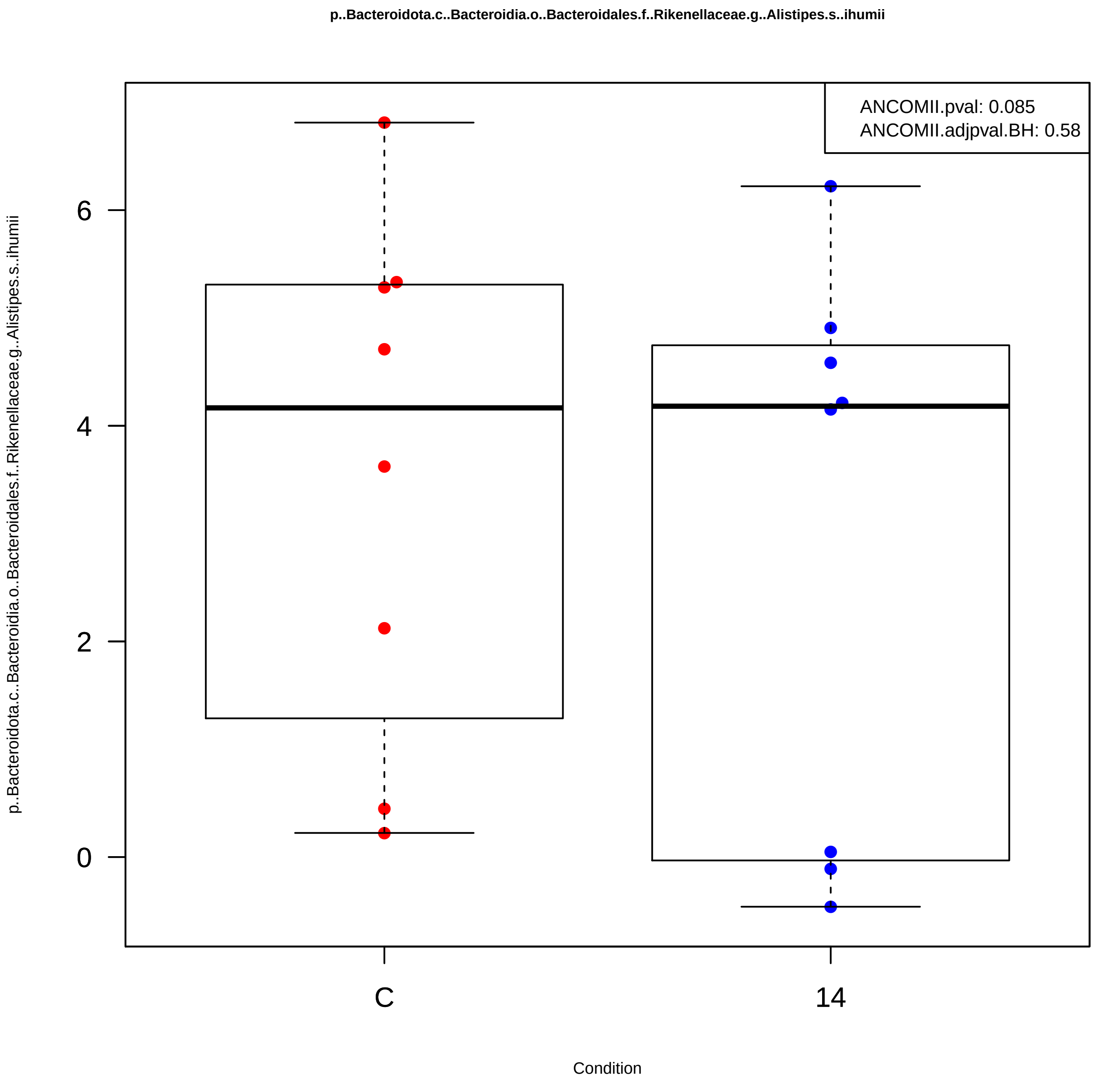

p..Bacteroidota.c..Bacteroidia.o..Bacteroidales.f..Rikenellaceae.g..Alistipes.s..ihumii
ANCOMII.pval: 0.085
ANCOMII.adjpval.BH: 0.58
p..Bacteroidota.c..Bacteroidia.o..Bacteroidales.f..Rikenellaceae.g..Alistipes.s..ihumii
6
4
2
0
C
14
Condition

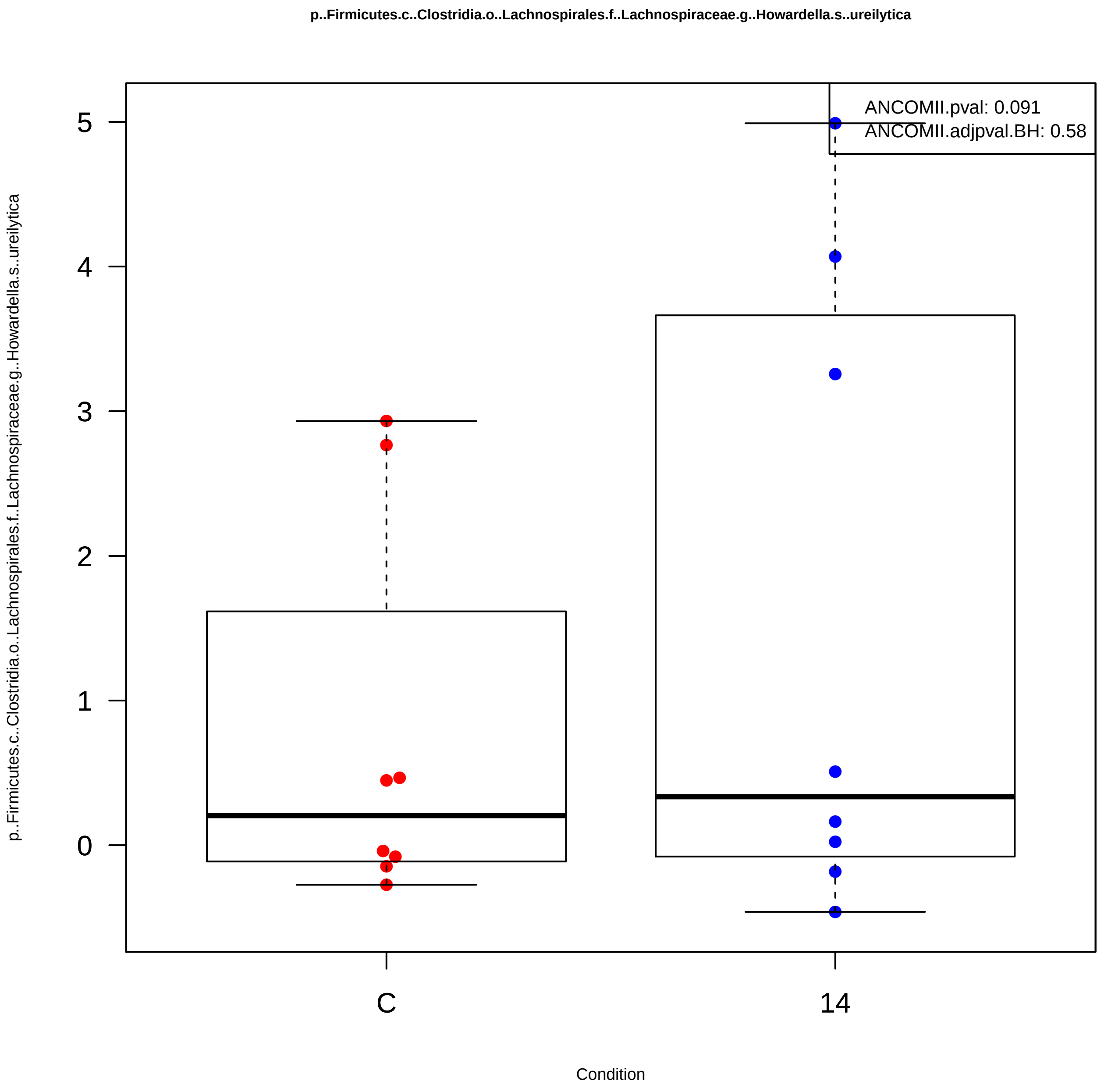
p..Firmicutes.c..Clostridia.o..Lachnospirales.f..Lachnospiraceae.g..Howardella.s..ureilytica
ANCOMII.pval: 0.091
ANCOMII.adjpval.BH: 0.58
p..Firmicutes.c..Clostridia.o..Lachnospirales.f..Lachnospiraceae.g..Howardella.s..ureilytica
0
1
2
3
4
5
C
14
Condition
