# Supplementary material for: Evaluation of the Effects of a Short Supplementation With Tannins on the Gut Microbiota of Healthy Subjects
Source: Front Microbiol. 2022 Apr 27;13:848611. doi: 10.3389/fmicb.2022.848611 (PMC9093706; doi:10.3389/fmicb.2022.848611)
Supplement: Supplementary file 5 [file Data_Sheet_5.PDF]

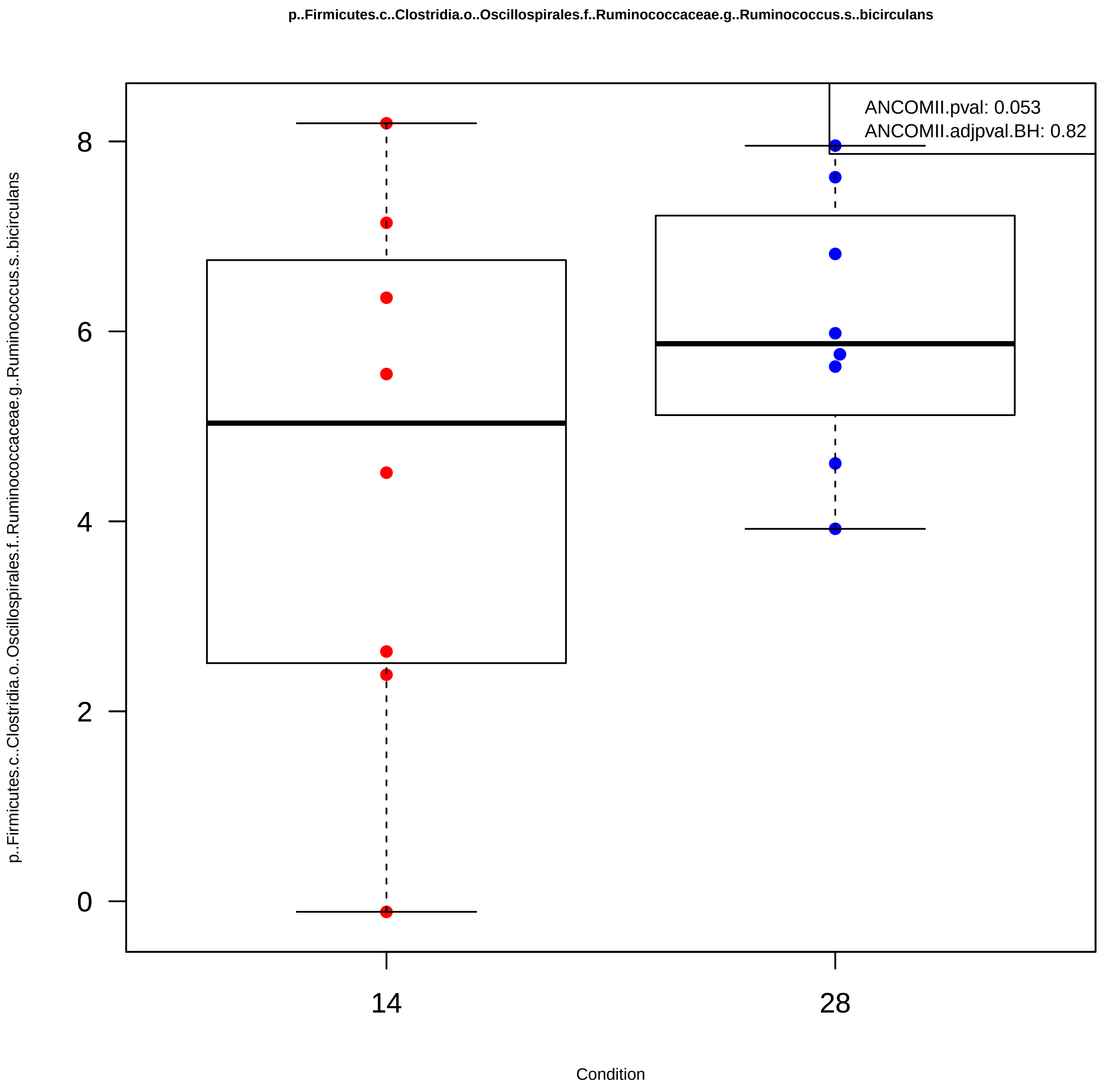
p..Firmicutes.c..Clostridia.o..Oscillospirales.f..Ruminococcaceae.g..Ruminococcus.s..bicirculans
ANCOMII.pval: 0.053
ANCOMII.adjpval.BH: 0.82
8
6
4
2
0
14
28
Condition
p..Firmicutes.c..Clostridia.o..Oscillospirales.f..Ruminococcaceae.g..Ruminococcus.s..bicirculans

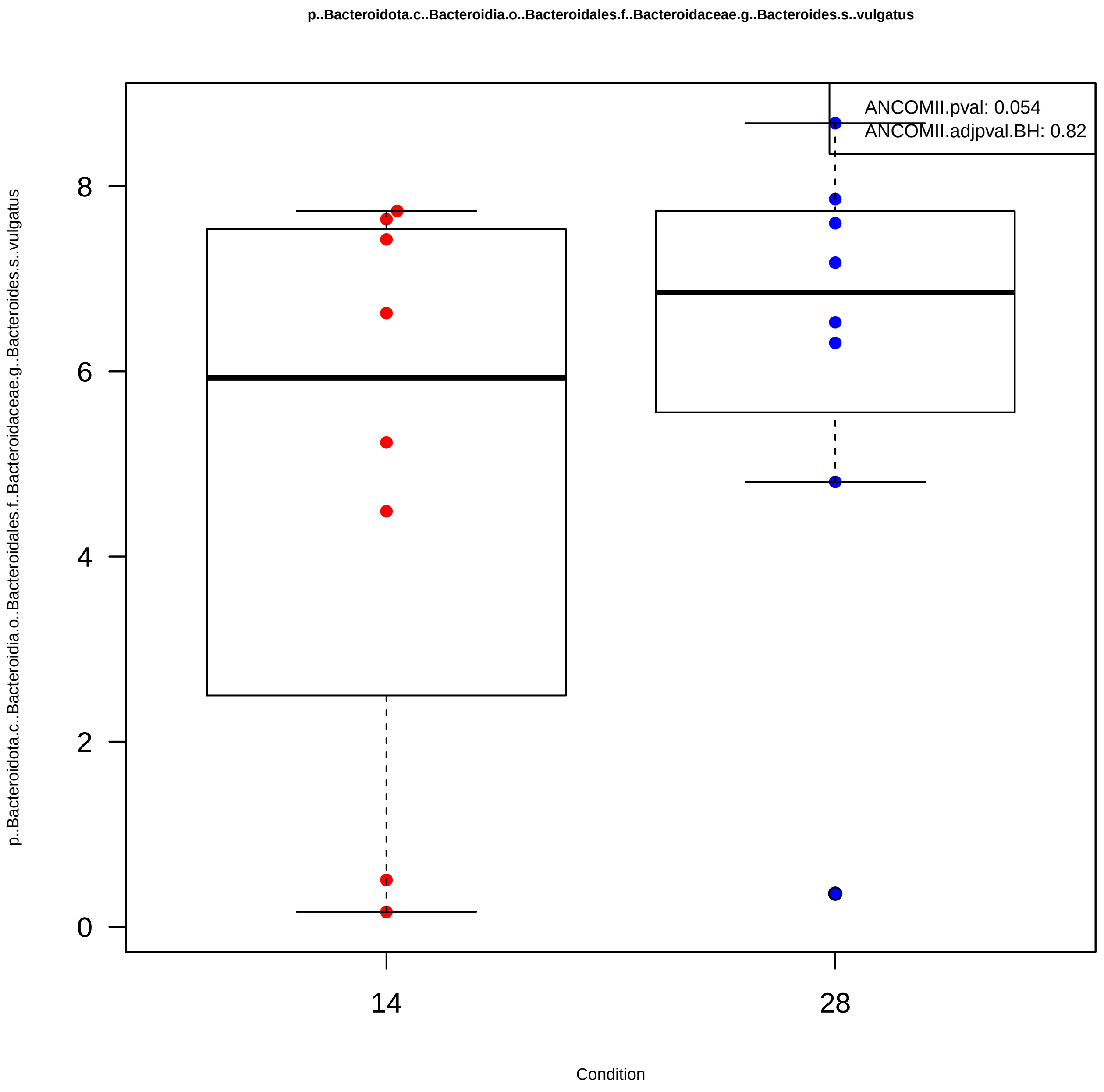

p..Bacteroidota.c..Bacteroidia.o..Bacteroidales.f..Bacteroidaceae.g..Bacteroides.s..vulgatus
ANCOMII.pval: 0.054
ANCOMII.adjpval.BH: 0.82
8
6
4
2
0
p..Bacteroidota.c..Bacteroidia.o..Bacteroidales.f..Bacteroidaceae.g..Bacteroides.s..vulgatus
14
28
Condition

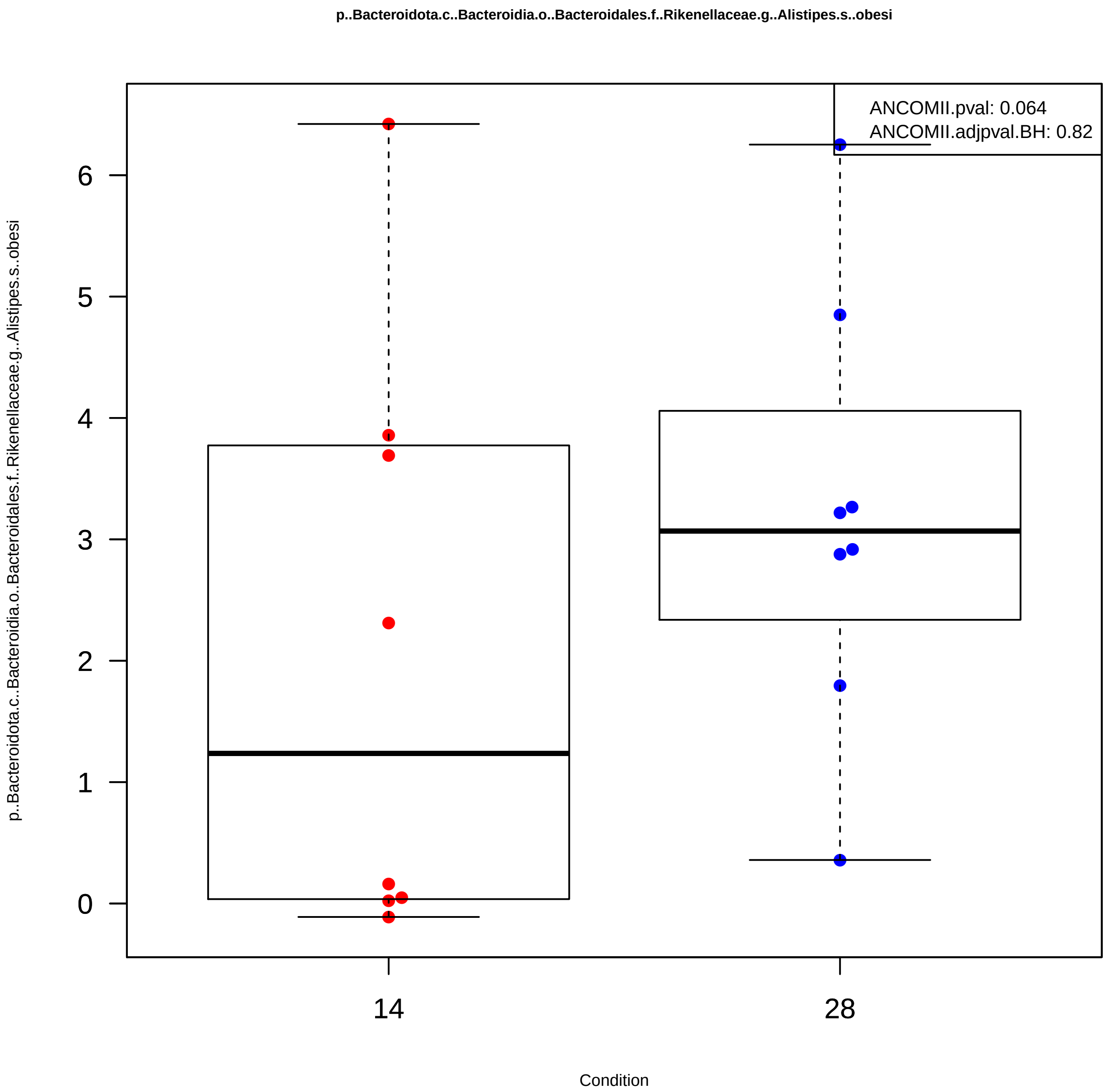

p..Bacteroidota.c..Bacteroidia.o..Bacteroidales.f..Rikenellaceae.g..Alistipes.s..obesi
ANCOMII.pval: 0.064
ANCOMII.adjpval.BH: 0.82
0
1
2
3
4
5
6
p..Bacteroidota.c..Bacteroidia.o..Bacteroidales.f..Rikenellaceae.g..Alistipes.s..obesi
14
28
Condition

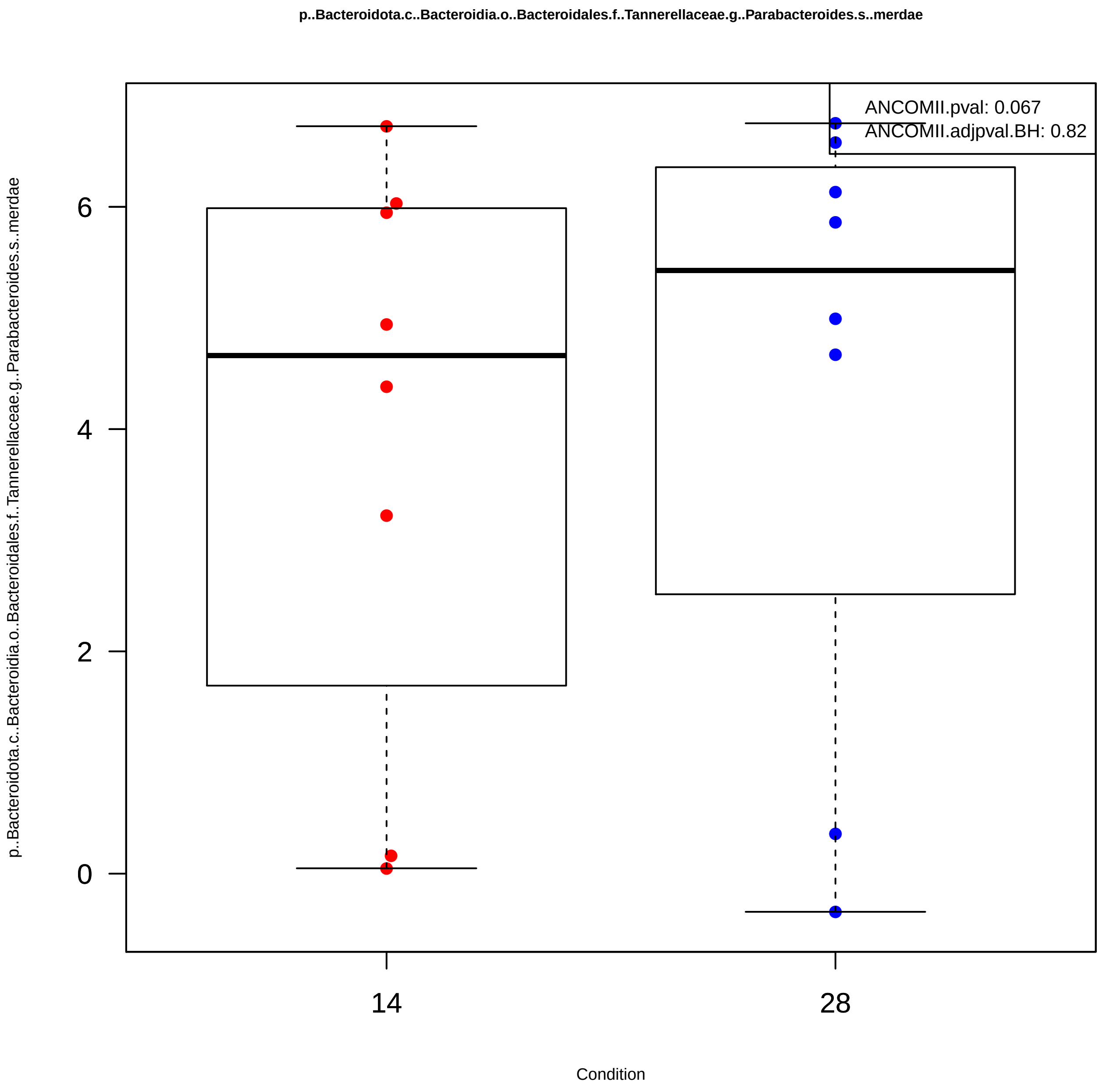

p..Bacteroidota.c..Bacteroidia.o..Bacteroidales.f..Tannerellaceae.g..Parabacteroides.s..merdae
ANCOMII.pval: 0.067
ANCOMII.adjpval.BH: 0.82
p..Bacteroidota.c..Bacteroidia.o..Bacteroidales.f..Tannerellaceae.g..Parabacteroides.s..merdae
0
2
4
6
14
28
Condition

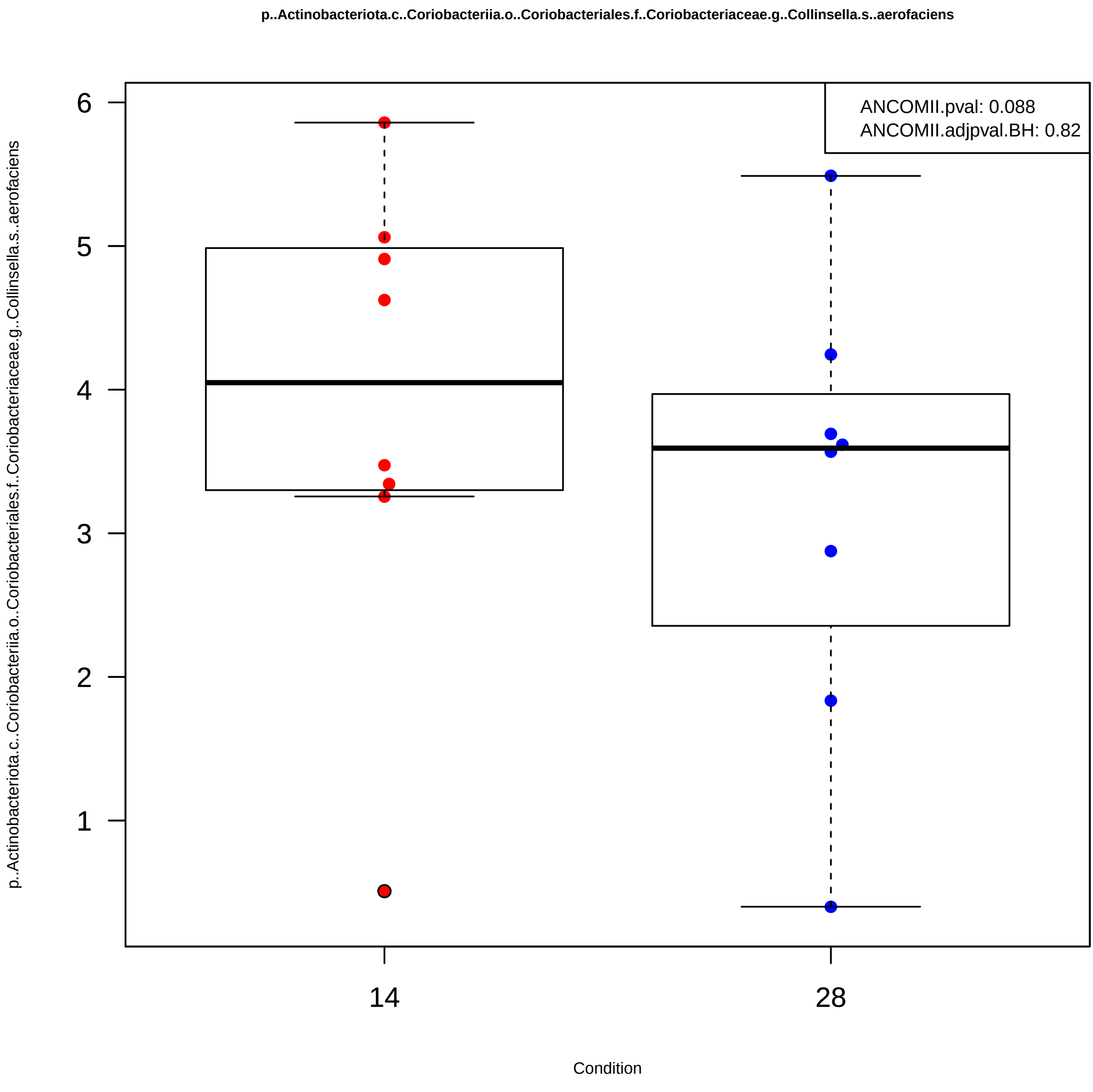

p..Actinobacteriota.c..Coriobacteriia.o..Coriobacteriales.f..Coriobacteriaceae.g..Collinsella.s..aerofaciens
ANCOMII.pval: 0.088
ANCOMII.adjpval.BH: 0.82
p..Actinobacteriota.c..Coriobacteriia.o..Coriobacteriales.f..Coriobacteriaceae.g..Collinsella.s..aerofaciens
6
5
4
3
2
1
14
28
Condition
