# Supplementary material for: Evaluation of the Effects of a Short Supplementation With Tannins on the Gut Microbiota of Healthy Subjects
Source: Front Microbiol. 2022 Apr 27;13:848611. doi: 10.3389/fmicb.2022.848611 (PMC9093706; doi:10.3389/fmicb.2022.848611)
Supplement: Supplementary file 6 [file Data_Sheet_6.PDF]

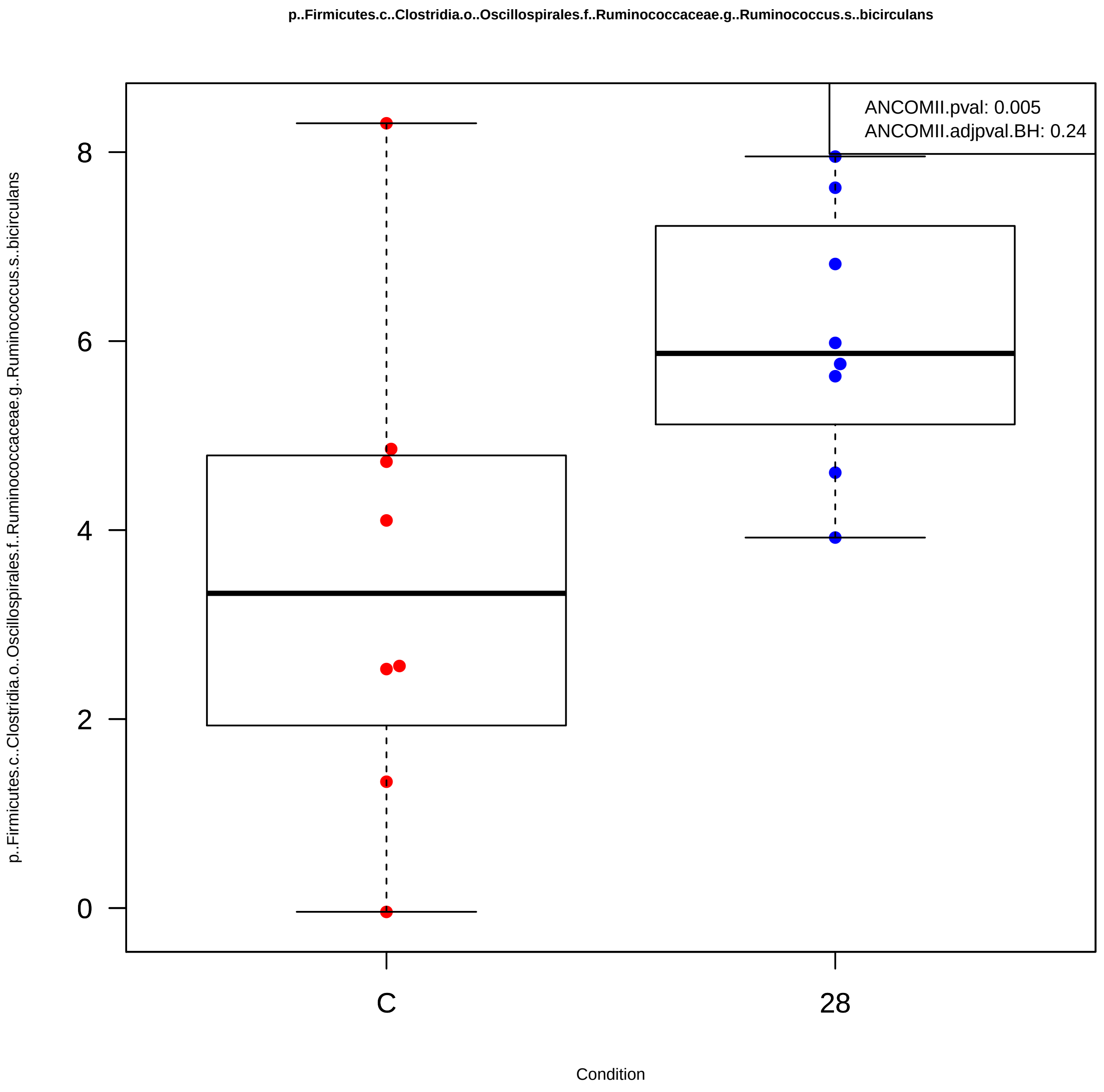

p..Firmicutes.c..Clostridia.o..Oscillospirales.f..Ruminococcaceae.g..Ruminococcus.s..bicirculans
ANCOMII.pval: 0.005
ANCOMII.adjpval.BH: 0.24
0
2
4
6
8
C
28
Condition
p..Firmicutes.c..Clostridia.o..Oscillospirales.f..Ruminococcaceae.g..Ruminococcus.s..bicirculans

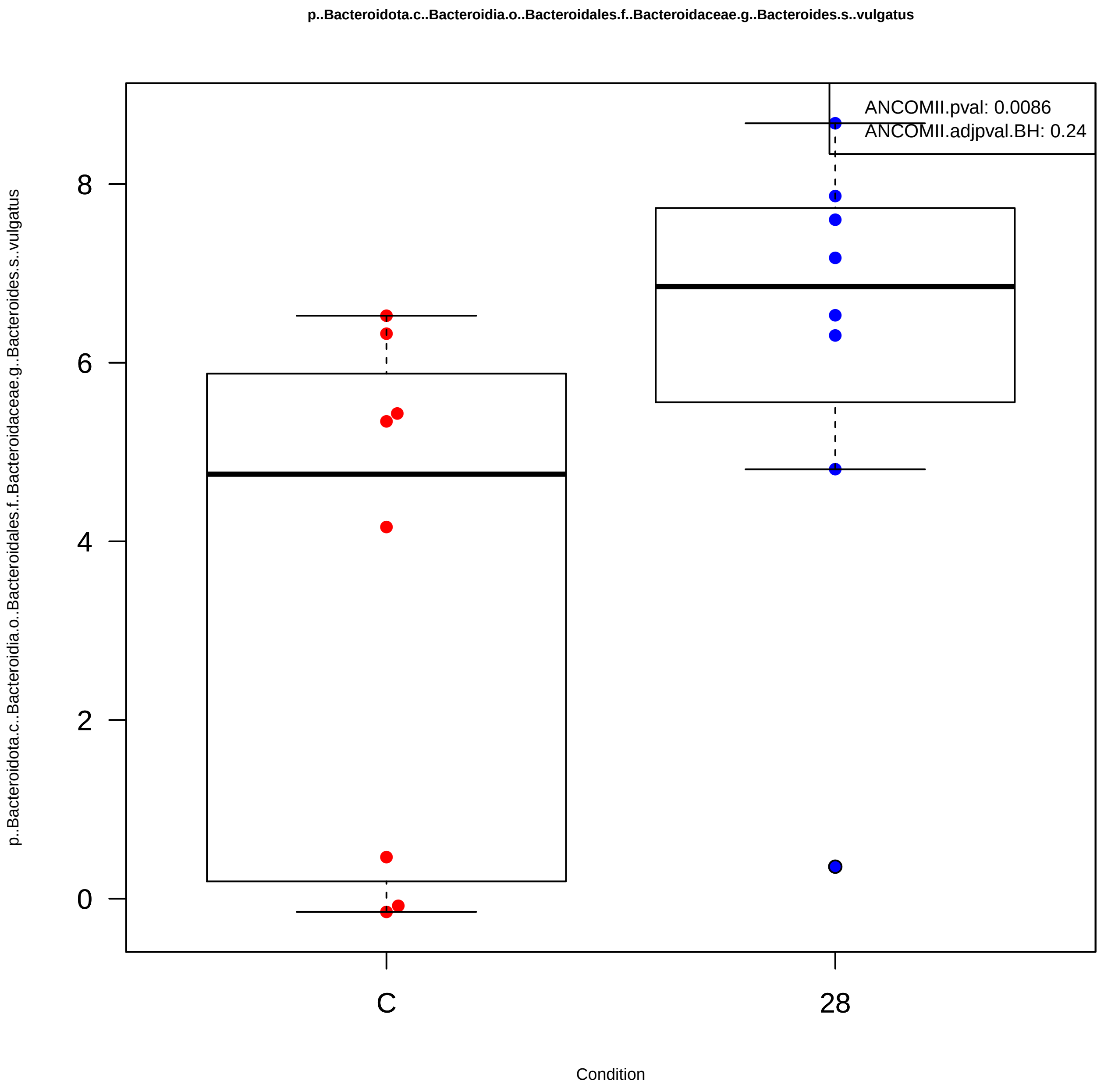

p..Bacteroidota.c..Bacteroidia.o..Bacteroidales.f..Bacteroidaceae.g..Bacteroides.s..vulgatus
ANCOMII.pval: 0.0086
ANCOMII.adjpval.BH: 0.24
0
2
4
6
8
C
28
Condition
p..Bacteroidota.c..Bacteroidia.o..Bacteroidales.f..Bacteroidaceae.g..Bacteroides.s..vulgatus

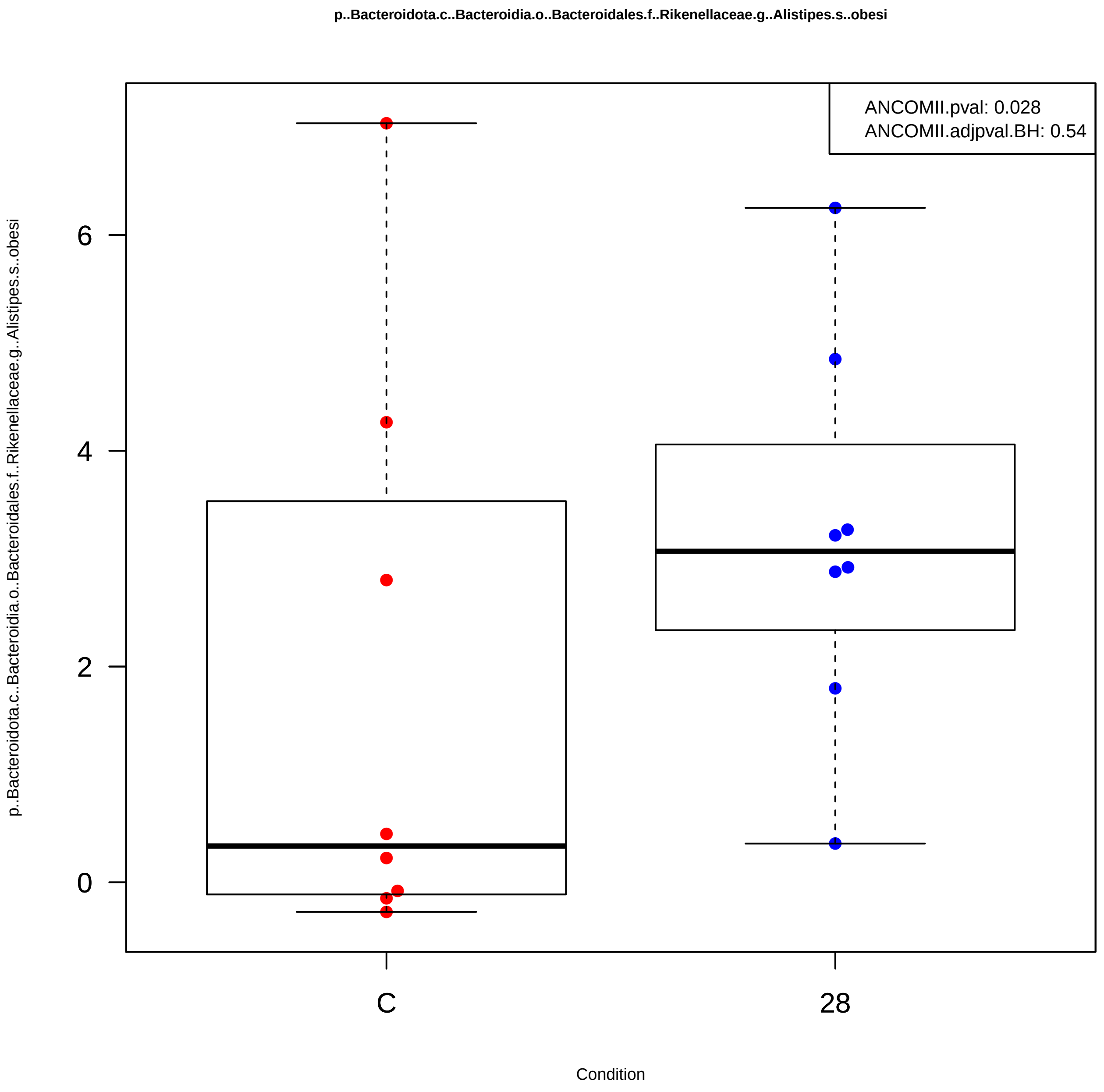
p..Bacteroidota.c..Bacteroidia.o..Bacteroidales.f..Rikenellaceae.g..Alistipes.s..obesi
ANCOMII.pval: 0.028
ANCOMII.adjpval.BH: 0.54
p..Bacteroidota.c..Bacteroidia.o..Bacteroidales.f..Rikenellaceae.g..Alistipes.s..obesi
0
2
4
6
C
28
Condition

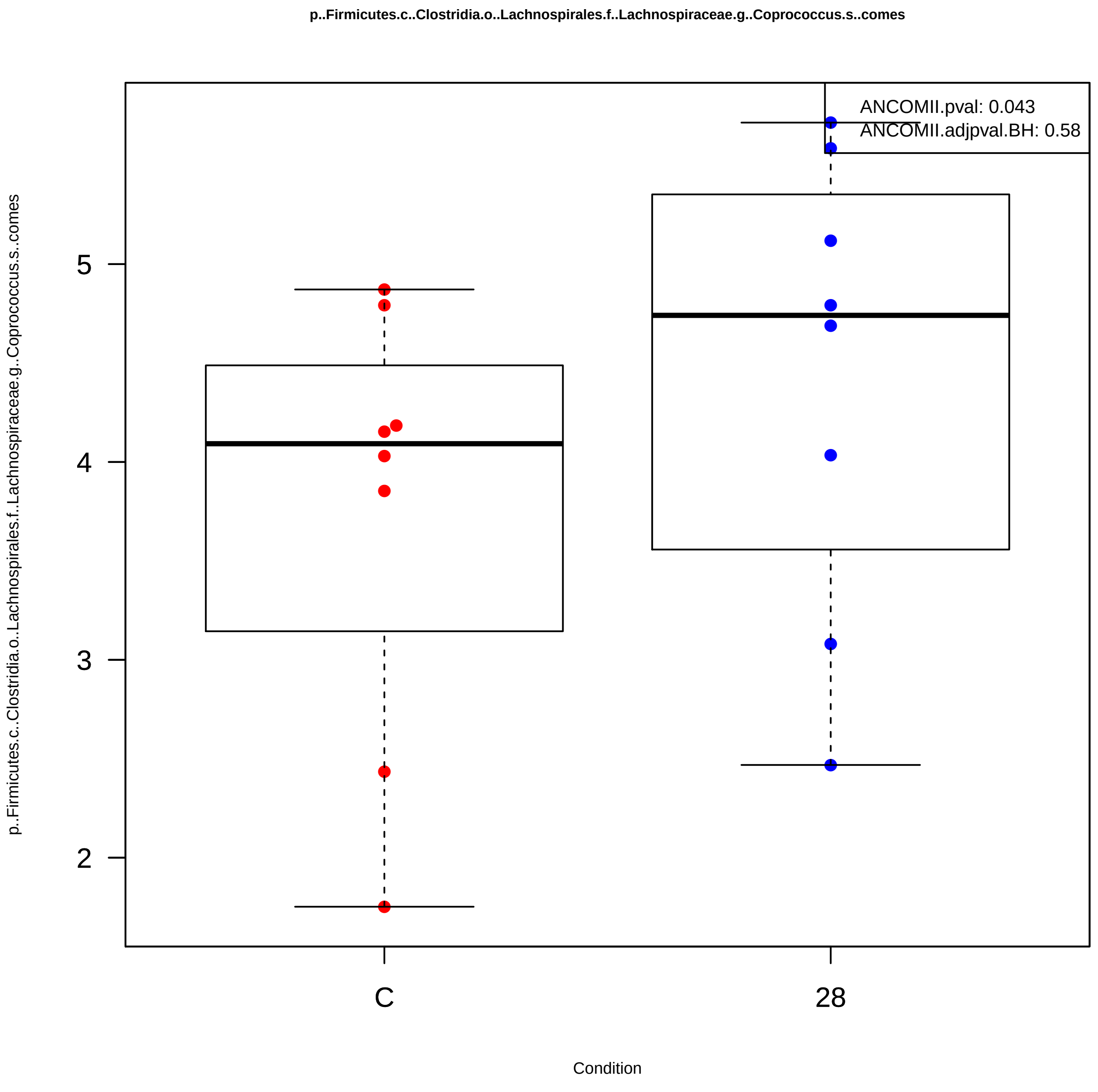

p..Firmicutes.c..Clostridia.o..Lachnospirales.f..Lachnospiraceae.g..Coprococcus.s..comes
ANCOMII.pval: 0.043
ANCOMII.adjpval.BH: 0.58
p..Firmicutes.c..Clostridia.o..Lachnospirales.f..Lachnospiraceae.g..Coprococcus.s..comes
5
4
3
2
C
28
Condition

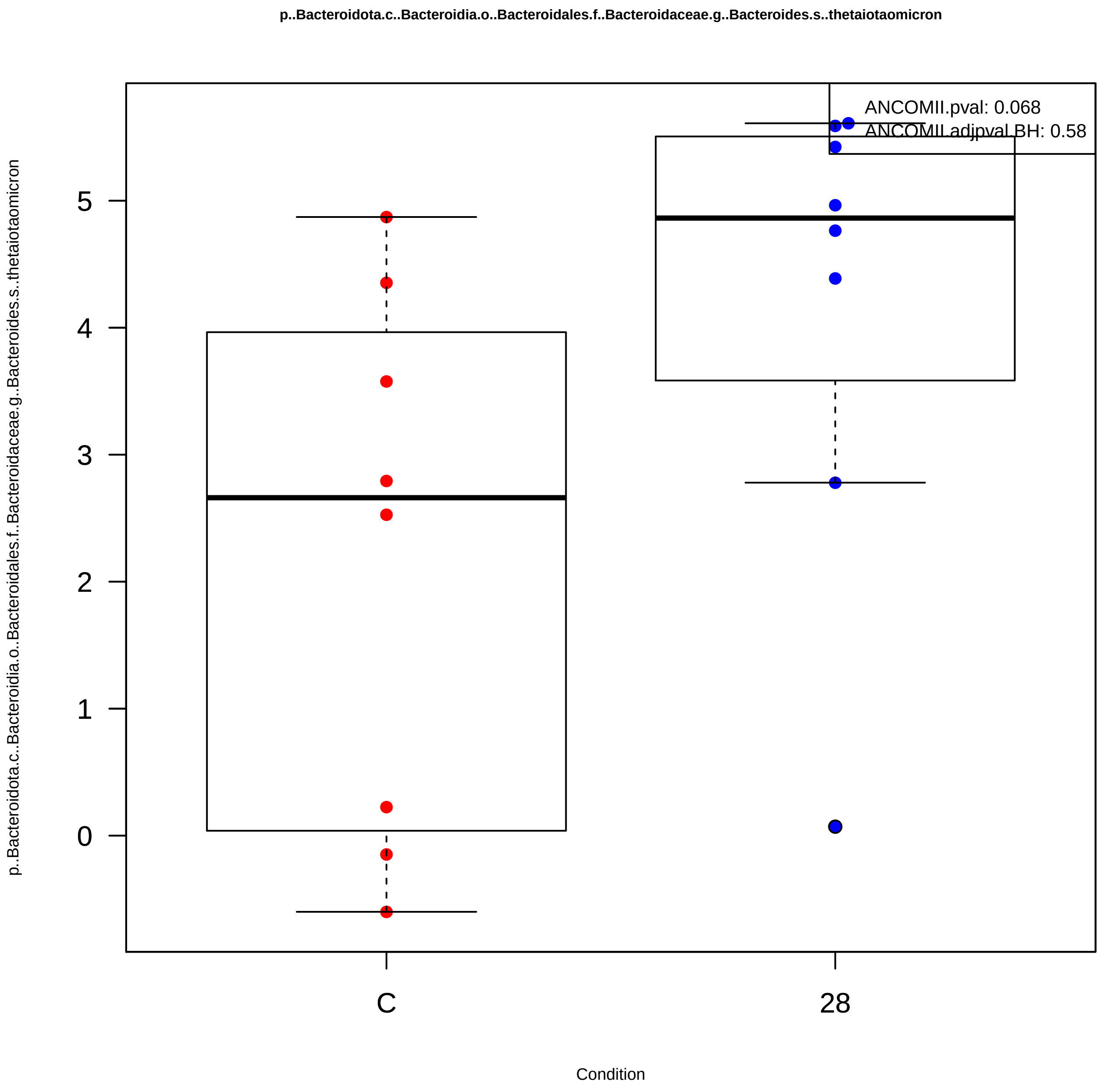

p..Bacteroidota.c..Bacteroidia.o..Bacteroidales.f..Bacteroidaceae.g..Bacteroides.s..thetaiotaomicron
ANCOMII.pval: 0.068
ANCOMII.adjpval.BH: 0.58
p..Bacteroidota.c..Bacteroidia.o..Bacteroidales.f..Bacteroidaceae.g..Bacteroides.s..thetaiotaomicron
0
1
2
3
4
5
C
28
Condition

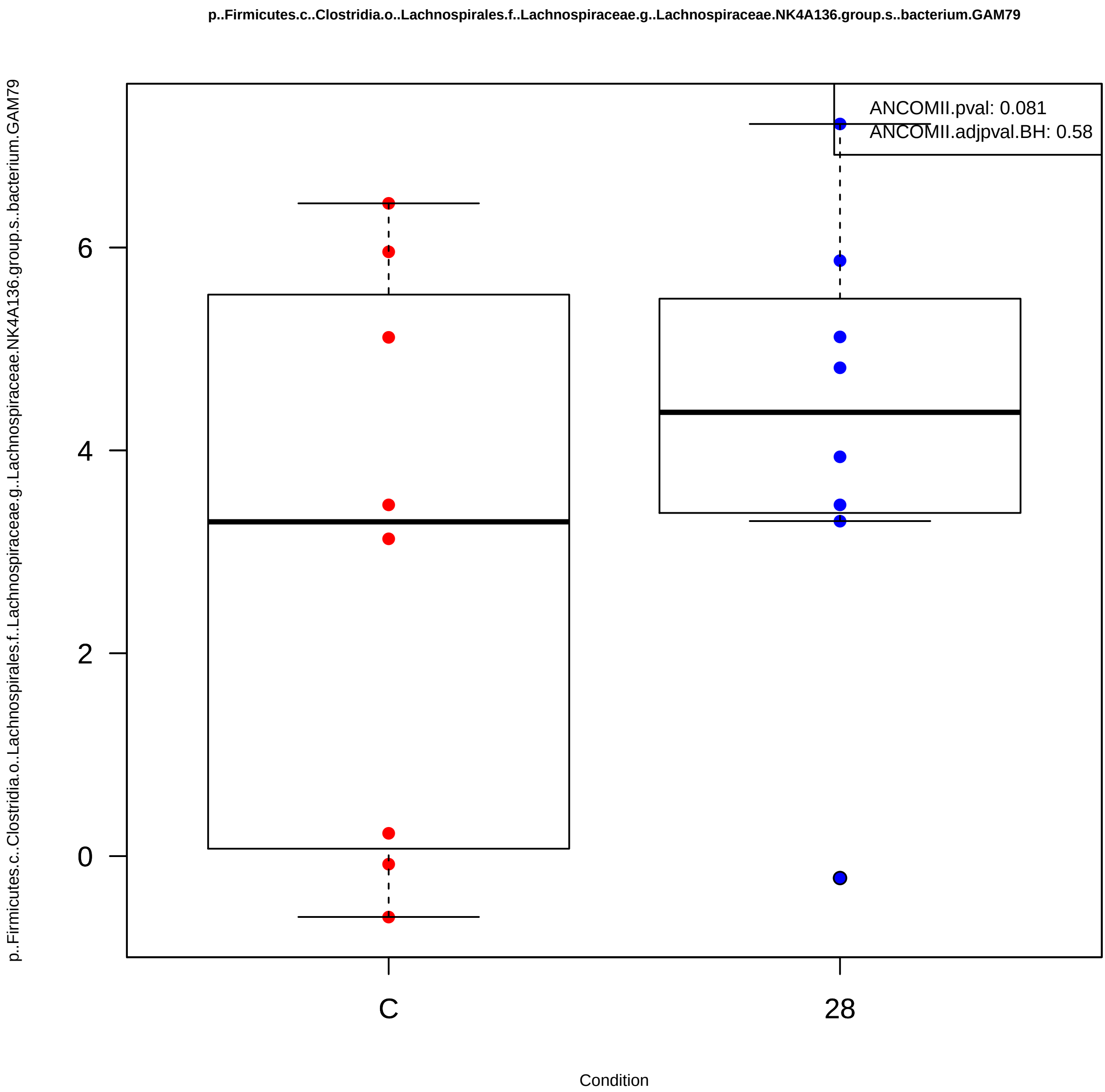
p..Firmicutes.c..Clostridia.o..Lachnospirales.f..Lachnospiraceae.g..Lachnospiraceae.NK4A136.group.s..bacterium.GAM79
ANCOMII.pval: 0.081
ANCOMII.adjpval.BH: 0.58
6
4
2
0
C
28
Condition
p..Firmicutes.c..Clostridia.o..Lachnospirales.f..Lachnospiraceae.g..Lachnospiraceae.NK4A136.group.s..bacterium.GAM79

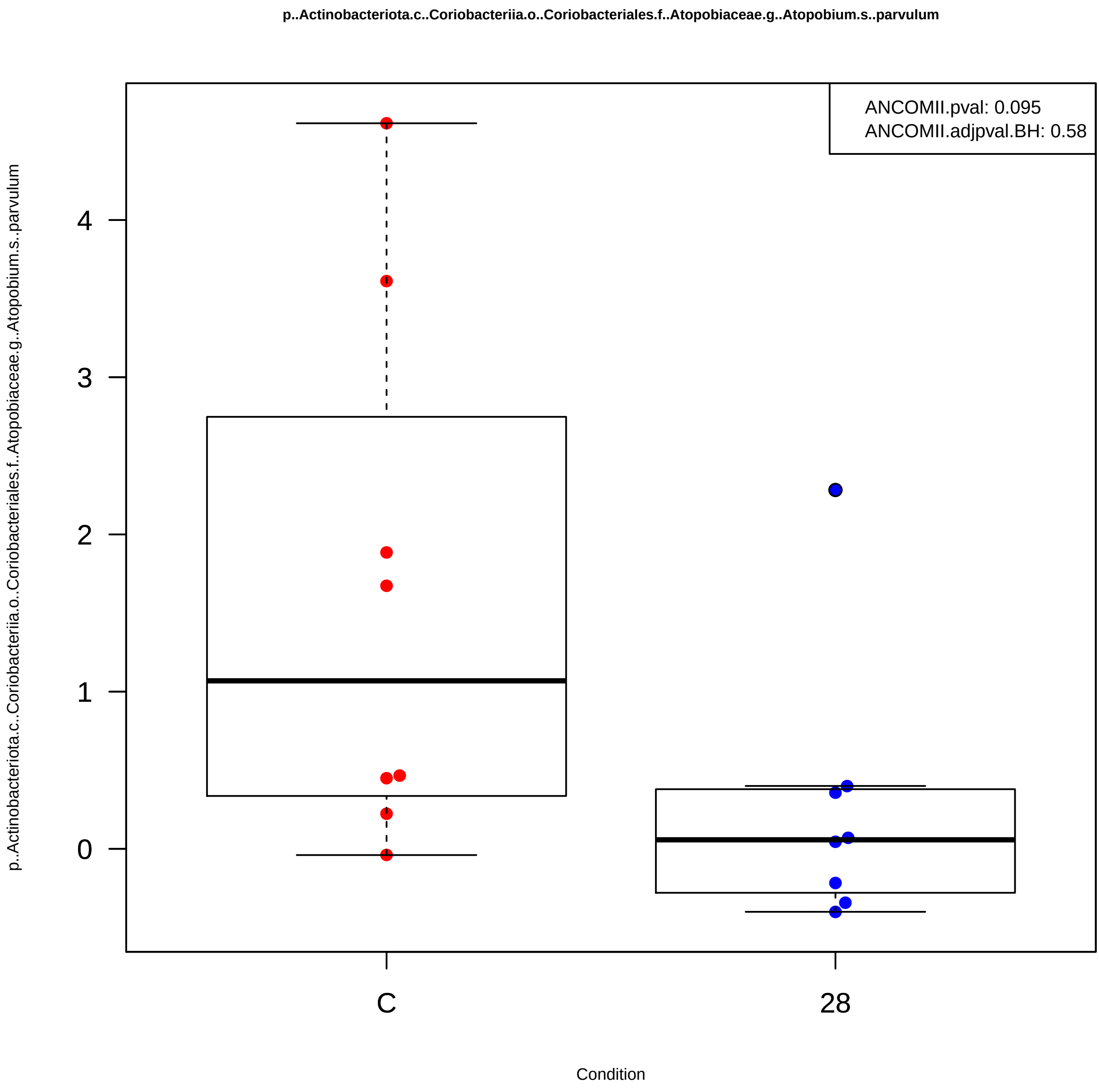
p..Actinobacteriota.c..Coriobacteriia.o..Coriobacteriales.f..Atopobiaceae.g..Atopobium.s..parvulum
ANCOMII.pval: 0.095
ANCOMII.adjpval.BH: 0.58
p..Actinobacteriota.c..Coriobacteriia.o..Coriobacteriales.f..Atopobiaceae.g..Atopobium.s..parvulum
4
3
2
1
0
C
28
Condition

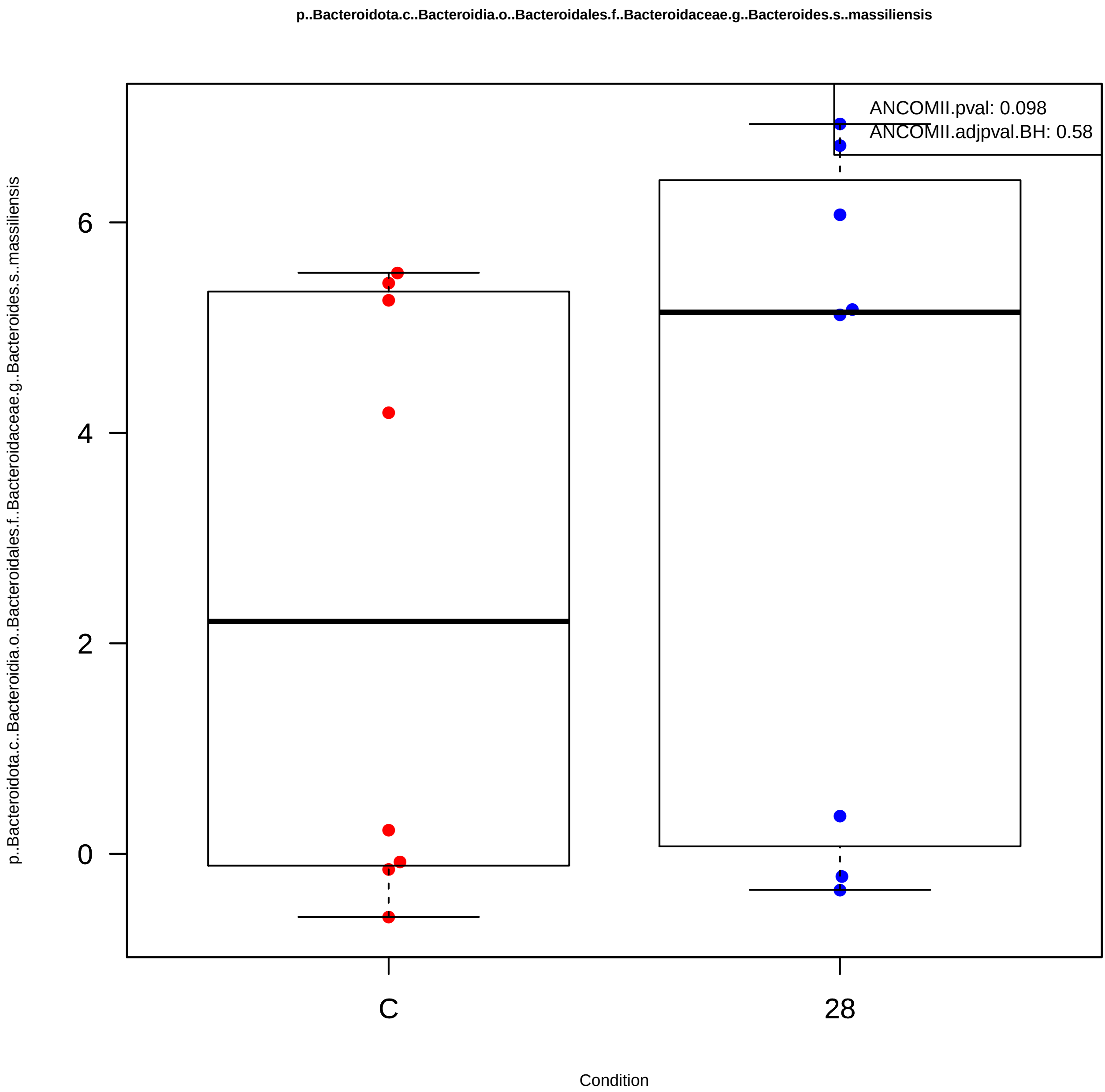
p..Bacteroidota.c..Bacteroidia.o..Bacteroidales.f..Bacteroidaceae.g..Bacteroides.s..massiliensis
ANCOMII.pval: 0.098
ANCOMII.adjpval.BH: 0.58
6
4
2
0
C
28
Condition
p..Bacteroidota.c..Bacteroidia.o..Bacteroidales.f..Bacteroidaceae.g..Bacteroides.s..massiliensis
