# Supplementary material for: Evaluation of the Effects of a Short Supplementation With Tannins on the Gut Microbiota of Healthy Subjects
Source: Front Microbiol. 2022 Apr 27;13:848611. doi: 10.3389/fmicb.2022.848611 (PMC9093706; doi:10.3389/fmicb.2022.848611)
Supplement: Supplementary file 7 [file Table_1.DOC]

Supplementary Material

# Evaluation of the effects of a short supplementation with tannins on the gut microbiota of healthy subjects

Silvia Molino^1^, Alberto Lerma-Aguilera^2^, Nuria Jiménez-Hernández^2^,3, José Ángel Rufián Henares^1,4*^, M. Pilar Francino^2,3*^

^1^Departamento de Nutrición y Bromatología, Instituto de Nutrición y Tecnología de los Alimentos, Centro de Investigación Biomédica, Universidad de Granada, Granada, Spain

^2^Area de Genòmica i Salut, Fundació per al Foment de la Investigació Sanitária i Biomèdica de la Comunitat Valenciana (FISABIO-Salut Pública), València, Spain

^3^ CIBER en Epidemiología y Salud Pública, Madrid, 28029, Spain

^4^ Instituto de Investigación Biosanitaria ibs.Granada, Granada, Spain

*** Correspondence:**M. Pilar Francino
[mpfrancino@gmail.com](mailto:mpfrancino@gmail.com)

José Ángel Rufián Henares
[jarufian@ugr.es](mailto:jarufian@ugr.es)

**1. Supplementary Table**

**Table S1**. Indexes of α diversity and relative abundance of phyla.

|  |  | **Diversity indexes** | | | | | **Phyla composition** | | | | | |
| --- | --- | --- | --- | --- | --- | --- | --- | --- | --- | --- | --- | --- |
| **Individual** | **Time** | Shannon | Chao1 | SE.Chao1 | ACE | SE.ACE | Actinobacteria | Bacteroidota | Firmicutes | Proteobacteria | Verrucomicrobia | other |
| 2 | T0 | 2.95 | 165 | 0.25 | 165.2 | 4.80 | 1.61 | 17.21 | 35.59 | 9.36 | 35.63 | 0.60 |
|  | T 14d | 3.79 | 172 | 0 | 172 | 3.70 | 2.14 | 11.35 | 78.63 | 0.16 | 3.67 | 4.05 |
|  | T 28d | 3.36 | 145 | 0 | 145 | 4.48 | 0.14 | 23.90 | 47.11 | 1.20 | 26.29 | 1.37 |
| 3 | T0 | 4.15 | 219 | 0 | 219 | 5.23 | 0.22 | 31.16 | 63.40 | 1.69 | 0.00 | 3.52 |
|  | T 14d | 4.19 | 230 | 0 | 230 | 5.45 | 0.83 | 37.04 | 55.20 | 2.51 | 0.03 | 4.38 |
|  | T 28d | 4.23 | 259.5 | 1.3 | 259.3 | 5.65 | 0.67 | 23.89 | 70.64 | 1.73 | 0.00 | 3.06 |
| 4 | T0 | 3.77 | 156 | 0 | 156 | 3.99 | 3.29 | 13.31 | 77.21 | 2.09 | 3.85 | 0.26 |
|  | T 14d | 4.24 | 186 | 0 | 186 | 3.48 | 0.84 | 35.96 | 61.57 | 1.22 | 0.36 | 0.05 |
|  | T 28d | 3.95 | 160 | 0 | 160 | 3.69 | 6.21 | 29.34 | 60.08 | 4.29 | 0.07 | 0.01 |
| 5 | T0 | 3.87 | 185 | 0.5 | 185.2 | 4.10 | 3.22 | 9.27 | 75.30 | 3.22 | 7.12 | 1.87 |
|  | T 14d | 3.42 | 173 | 0.25 | 173.2 | 4.74 | 1.01 | 22.04 | 51.87 | 0.67 | 23.78 | 0.63 |
|  | T 28d | 4.08 | 178 | 0 | 178 | 4.39 | 0.50 | 38.09 | 54.60 | 1.22 | 5.24 | 0.36 |
| 6 | T0 | 3.48 | 152 | 0 | 152 | 4.78 | 0.31 | 17.77 | 62.28 | 2.94 | 15.26 | 1.44 |
|  | T 14d | 4.10 | 216 | 0 | 216 | 4.94 | 1.48 | 22.38 | 71.18 | 0.73 | 3.48 | 0.74 |
|  | T 28d | 3.95 | 217 | 0.25 | 217.2 | 4.76 | 0.28 | 27.15 | 57.82 | 2.42 | 11.50 | 0.83 |
| 10 | T0 | 3.01 | 104 | 0 | 104 | 3.26 | 0.73 | 40.80 | 37.38 | 1.27 | 19.74 | 0.08 |
|  | T 14d | 3.06 | 110 | 0 | 110 | 3.15 | 0.95 | 41.60 | 35.01 | 2.07 | 20.15 | 0.21 |
|  | T 28d | 3.17 | 128 | 0 | 128 | 3.17 | 0.54 | 40.96 | 35.58 | 3.35 | 19.19 | 0.39 |
| 11 | T0 | 4.12 | 207 | 0 | 207 | 4.92 | 2.16 | 19.88 | 66.73 | 11.10 | 0.02 | 0.11 |
|  | T 14d | 4.23 | 213 | 0.25 | 213.2 | 4.98 | 1.48 | 37.43 | 59.16 | 1.60 | 0.08 | 0.25 |
|  | T 28d | 4.25 | 242.3 | 0.93 | 242.5 | 5.30 | 2.08 | 40.96 | 54.72 | 0.86 | 1.28 | 0.09 |
| 13 | T0 | 3.39 | 168 | 0 | 168 | 4.37 | 2.73 | 24.42 | 71.95 | 0.50 | 0.00 | 0.40 |
|  | T 14d | 4.01 | 195 | 0 | 195 | 4.75 | 2.97 | 31.91 | 61.64 | 3.15 | 0.00 | 0.33 |
|  | T 28d | 4.25 | 212 | 0 | 212 | 4.93 | 2.16 | 34.86 | 62.26 | 0.35 | 0.00 | 0.37 |
|  |  |  |  |  |  |  |  |  |  |  |  |  |

**2. Supplementary Figures**

**Figure S1** Comparisons of relative abundances of bacteria at family level: T0 vs T14d (S1A), T14d vs T28d (S1B) and T0 vs T28d (S1C). Adjusted and unadjusted p values by ANCOM tests are shown. (3 pdf files uploaded separately)

**Figure S2** Comparisons of relative abundances of bacteria at species level: T0 vs T14d (S2A), T14d vs T28d (S2B) and T0 vs T28d (S2C). Adjusted and unadjusted p values by ANCOM tests are shown. (3 pdf files uploaded separately)
